# *Rhodnius* (Stål, 1859) (Hemiptera, Triatominae) genus in Bolivian Amazonia: a risk for human populations?

**DOI:** 10.1186/s13071-022-05423-3

**Published:** 2022-08-29

**Authors:** Stéphanie Depickère, Anita G. Villacís, Soledad Santillán-Guayasamín, Jorgia Esperanza Callapa Rafael, Simone Frédérique Brenière, Susana Revollo Zepita

**Affiliations:** 1 Instituto de Investigaciones Físicas, Universidad Mayor de San Andrés, La Paz, Bolivia; 2 Centro de Investigación para la Salud en América Latina (CISeAL), Escuela de Ciencias Biológicas, Facultad de Ciencias Exactas y Naturales, Pontificia Universidad Católica del Ecuador, Quito, Ecuador; 3 Laboratorio de Genética Molecular (SELADIS), Facultad Ciencias Farmacéuticas y Bioquímicas, Universidad Mayor de San Andrés, La Paz, Bolivia; 4 Institut de Recherche pour le Développement (IRD), UMR INTERTRYP IRD-CIRAD, University of Montpellier, Montpellier, France

**Keywords:** Chagas, Triatominae, *Rhodnius stali*, *Rhodnius montenegrensis*, *Trypanosoma cruzi*, DTUs, Blood meal origin, Bolivia, Amazonia, Geographic distribution

## Abstract

**Background:**

Chagas disease, one of the most important neglected tropical diseases in the countries of Latin America, is considered to be a particularly important public health concern in the Amazon region due to increases in the number of outbreaks of acute Chagas disease and increased local transmission in the last 20 years. However, relative to other countries, in Bolivia there is little information available on its transmission in the Amazon region. The aim of this study was to investigate the infestation of palm trees, the main habitat of Triatominae in the region, in several localities, to evaluate the danger they represent to inhabitants.

**Methods:**

Triatominae were collected using live bait traps left overnight in six localities in Pando and Beni Departments, Bolivia. DNA extraction and sequencing were used to establish the Triatominae species (*Cytb*, *16S* and *28S-D2* gene fragments), and the blood meal sources (*Cytb* fragment). *Trypanosoma* sp. infection was analyzed by sequencing gene fragments (*GPX*, *GPI*, *HMCOAR*, *LAP*, *PDH* and *COII*) or by mini-exon multiplex PCR.

**Results:**

A total of 325 *Rhodnius* were captured (97.3% of nymphs) from the 1200 traps placed in 238 palm trees and 32 burrows/ground holes. Sequence analyses on DNA extracted from 114 insects and phylogeny analysis identified two triatomine species: *Rhodnius stali* (17%) and *Rhodnius montenegrensis* (equated to *Rhodnius robustus* II, 83%). These were found in palm trees of the genera *Attalea* (69%), *Astrocaryum* (13%), *Copernicia* (12%), *Euterpe* (2%) and *Acrocomia* (1%). The infection rate was around 30% (165 analyzed insects), with 90% of analyzed insects infected by *Trypanosoma cruzi* (only the TcI discrete typing unit was detected), 3% infected by *Trypanosoma rangeli* (first time found in Bolivian Triatominae) and 7% infected by mixed *T. cruzi* (*TcI*)-*T. rangeli*. *Rhodnius* specimens fed on Didelphidae, rodents, gecko and humans.

**Conclusions:**

The results of this study highlight the epidemiological importance of *Rhodnius* in the Bolivian Amazon region. The huge geographical distribution of *Rhodnius* and their proximity to the human dwellings, high infection rate and frequent meals on the human population highlight a risk of transmission of Chagas disease in the region.

**Graphical Abstract:**

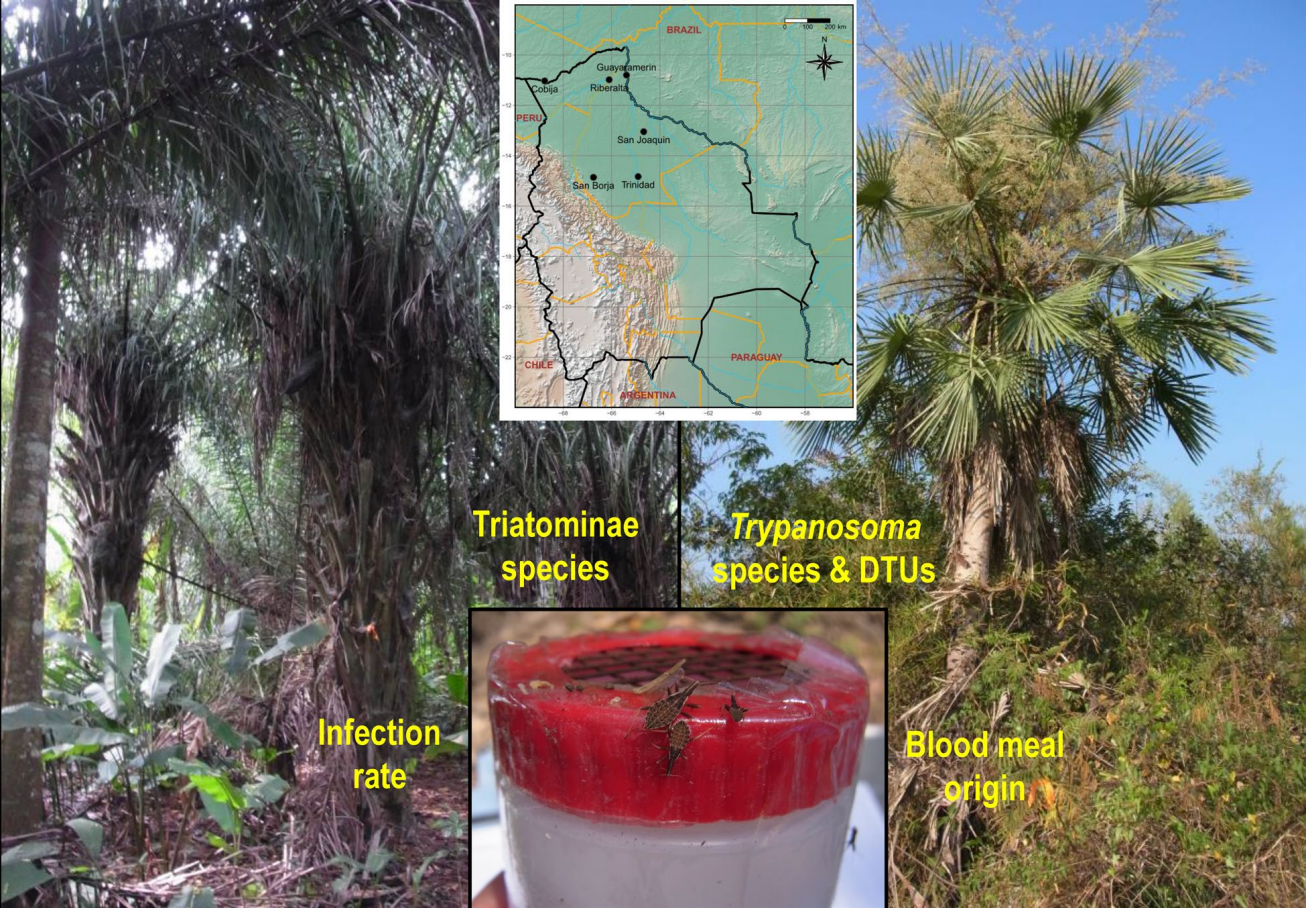

**Supplementary Information:**

The online version contains supplementary material available at 10.1186/s13071-022-05423-3.

## Background

Chagas disease remains one of the most important neglected diseases in the countries of Latin America. It is estimated that 6–7 million people are infected worldwide [1]. The disease is caused by a parasite, *Trypanosoma cruzi* (Chagas, 1909), which is mainly transmitted by hematophagous insects of the subfamily Triatominae (Hemiptera: Reduviidae). Other less frequent modes of transmission include congenital transmission, transfusion of contaminated blood products, transplantation of infected organs and oral transmission through ingestion of food or drinks contaminated with infected triatomine feces [1].

The Amazon region has long been considered a non-endemic region, despite the general belief that *T. cruzi* has circulated in the sylvatic environment for millions of years [2]. The first human case of Chagas disease in this region was reported in French Guiana in 1941 [2]. Since then, acute cases of Chagas disease have been diagnosed in almost all Pan-Amazonian countries [2]. In 2004, the Amazon Countries Initiative (AMCHA) was launched to monitor and prevent the disease in the nine countries sharing the Amazon rainforest: Bolivia, Brazil, Colombia, Ecuador, Guyana, French Guiana, Peru, Surinam and Venezuela [3]. An international workshop held in 2002 concluded that although disease occurrence remains sporadic, there is a high potential for spread due to the wide distribution of infected vectors in the region and intensive human migration [4]. In Brazil, national surveys and other studies have reported various outbreaks of acute Chagas disease in the Amazon region since 1975, mostly due to oral transmission [2]. The risks of endemic Chagas disease in the Amazon region are: (i) presence of a huge reservoir of wild mammals infected with *T. cruzi*; (ii) the presence of Triatominae found naturally infected with *T. cruzi*; (iii) the occurrence of acute oral Chagas disease transmission; (iv) uncontrolled deforestation; (v) the increasing migration of people and their domestic animals from other Chagas disease endemic regions; (vi) the lack of knowledge of Chagas disease transmission; and (vii) the poor surveillance campaigns in the region [2]. Despite the efforts and support of the Pan American Health Organization, very little has been done to fulfill the goals of the AMCHA [3].

In Bolivia, 60% of the territory is endemic to Chagas disease due to household infestation by the main vector *Triatoma infestans* (Klug, 1834). Since 1999, the authorities have prioritized the control of Chagas disease by focusing on this vector, using such strategies as insecticide spraying, screening blood donations and providing treatment for all acute cases and infected children under the age of 15 years [5]. The Amazon region of Bolivia represents 36–40% of the country’s territory and extends completely across the two departments of Beni and Pando and partly across the departments of La Paz, Cochabamba and Santa Cruz [5–7]. The Amazon region has been considered to date to be a non-endemic area for Chagas disease [5]. However, at least seven species of Triatominae have been reported to live in this region, namely *Rhodnius pictipes* (Stål, 1872), *Rhodnius robustus* (Larousse, 1927), *Rhodnius stali* (Lent, Jurberg & Galvão, 1993), *Panstrongylus geniculatus* (Latreille, 1811), *Microtriatoma trinidadensis* (Lent, 1951) and *Eratyrus mucronatus* (Stål, 1859), with some of these found in peridomestic and domestic environments [8–15]. In these areas, palm trees are known to be epidemiologically important in Chagas disease; in addition to providing a refuge for many mammals and birds, they also have the potential to contain large numbers of Triatominae, mostly of genus *Rhodnius* [16–18]. Palms are often of great importance to the local human population who used them mainly for house construction, food and handicrafts. These palms can be found throughout wild forest and near dwellings.

Very little is known about Chagas disease in the Bolivian Amazon region in terms of the presence and abundance of triatomine species or the infection rate of the human population. In the Amazonian part of the department of La Paz, a process of domiciliation of *R. stali* has been described in the peridomicile, and local transmission of Chagas disease to the human population has been detected [10, 13, 19]; this species has also been captured in palms in the same region [11]. An outbreak of acute Chagas disease by oral transmission has been reported in Beni Department, municipality of Guayaramerín in 2010, as a result of the ingestion of a palm fruit juice (*Oenocarpus bataua*; Majo in Bolivian) [19, 20]. A study in a small town at the foot of the Andes (Beni Department, on the border with La Paz Department) showed the presence of *R. robustus* and probably *R. stali* in palms [21]. Finally, in the tropics region of Cochabamba (Municipality of Villa Tunari), *P. geniculatus* (16 specimens) and *R. robustus* (5 specimens, species determined only by morphological examination) were found mainly in intra- and peridomiciles; 57% of these were infected *by T. cruzi* [22].

The identification of Triatominae, and in particular of *Rhodnius* species, is currently the subject of intense research, as many species belong to cryptic species complexes [23]. Three groups of *Rhodnius* are generally described: *pictipes* (7 species), *prolixus* (11 species) and *pallescens* (3 species) [24, 25]. Within the* prolixus* group, *R. prolixus* (Stål, 1859), *R. robustus*, *R. montenegrensis* (da Rosa et al., 2012) and* R. marabaensis* (Souza et al., 2016) are very close morphologically (sibling species, or pseudo-cryptic species, see [26]) and are generally grouped under the compound term of “*R. prolixus*–*R. robustus* cryptic-species complex” [27]. This complex is currently under study, and a recent publication has characterized all five nymphal instars and established the differences between *R. prolixus*, *R. robustus* and *R. marabaensis* in each of their instars [28]. Studies have shown that *R. prolixus* is a monophyletic group whereas *R. robustus* sensu lato appears to be paraphyletic with five subclades noted I to V [23, 29, 30]. Recently, the paraphyletic lineage *R. robustus* II was described as *R. montenegrensis* [27]. *Rhodnius taquarussuensis* (da Rosa et al. 2017) is now considered a phenotype of *R. neglectus* (Lent, 1954) [31]. Another relevant complex of *Rhodnius* species in Bolivia is the *R. pictipes* complex, composed of *R. amazonicus* (Almeida, Santos & Sposina, 1973), *R. paraensis* (Sherlock, Guitton & Miles, 1977), *R. zeledoni* (Jurberg, Rocha & Galvão, 2009), *R. pictipes*, *R. stali*, *R. brethesi* (Matta, 1919) and *R. micki* (Zhao, Galvão & Cai, 2021), the latter being a newly described species from Bolivia [24].

The aim of the present study was to increase basic entomological knowledge of Chagas disease in the Bolivian departments of Beni and  Pando and to obtain a preliminary assessment of the risk that wild Triatominae can represent in this region. These two departments cover a vast area of approximately 278,000 km^2^. Six cities were selected as study sites, and the exploration for Triatominae was done by passive searching of palms using traps. In parallel to this study, a survey on the incidence of Chagas disease in the population of these six cities was carried out in collaboration with the representative of the Amazonian Indigenous People, with the results showing that between 2% and 12% of the population had positive serological test results for Chagas disease [32].

## Methods

### Area description

The department of  Pando, located in the north of Bolivia, on the border with Brazil and Peru, in the Amazon rainforest, receives between 1500 and 2100 mm of rain per year (https://www.worldwildlife.org/ecoregions/nt0166). The department has a surface area of 63,827 km^2^, and a population of 110,000 inhabitants. The department of Beni is located in the country’s frequently flooded lowlands area and is characterized by the presence of savannas. The countryside shows a succession of forest fragments and pastures, with scattered ranches. Beni has a surface area of 213,564 km^2^, and a population of 420,000 inhabitants. In both departments, poverty levels are high, and access is difficult, especially during the rainy season. According to Census 2012 (https://www.ine.gob.bo/index.php/censos-y-banco-de-datos/censos/), the houses are typically made of bricks (46 ± 17%) or wood (34 ± 14%) walls, with dirt (46 ± 22%) or cement/tiled (42 ± 8%) floors, and with roofs of sheet metal (42 ± 29%), tiles (37 ± 28%) or palms (15 ± 14%).

### Selection of capture locations

**Fig. 1:**
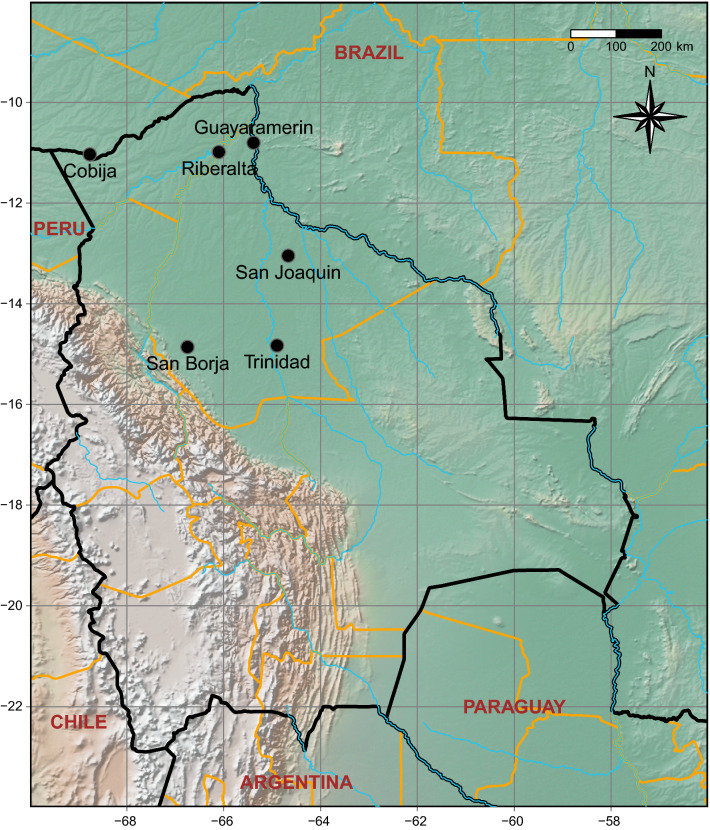
Map of Bolivia, with the location of the six cities chosen as capture locations for triatomines. Country borders are shown in black and department delimitations are shown in orange, the rivers being in blue. The map produced with Natural Earth and R software [36]

The presence of triatomines was investigated in six different locations during two different trips. The cities of Cobija, Riberalta and Guayaramerín were surveyed in August 2016, and those of Trinidad, San Borja and San Joaquin were surveyed in August 2017 (Fig. 1). All six cities are situated at an altitude falling within the range of 140–280 m a.s.l. Trinidad (capital of Beni department) has the highest number of inhabitants (125,000), followed by Riberalta (99,000), Cobija (capital of Pando department, 56,000), Guayaramerín (39,000), San Borja (25,000) and San Joaquin (4500).

For each location, three capture points (only 2 in Guayaramerín) were selected within a 30-km radius of the city. These were named using the initial of the city (SB: San Borja, SJ: San Joaquin, T: Trinidad, C: Cobija, G: Guayaramerín, R: Riberalta) and a number ranging from 1 to 3. These points were characterized by the presence of palms and accessibility to our team (no flooding and permission of local health authorities and/or owners). The environment of each capture point was described based on Google Earth photos using a circle of 500 m in radius around the capture point. The categories of land use were crops, forest, water, pasture/herbaceous, pasture/herbaceous with isolated palms, road, construction, open building plot and others. This last category comprised vacant land, land without vegetation and peridomiciles. The area of each category for each capture point was calculated (see details of surfaces and illustration of each capture point in Additional file 1: Figure S1). A principal component analysis (PCA) was realized with all of the capture points and land use categories using the *psych* [33], *FactoMineR* [34] and *ggrepel* [35] packages in R software [36]. The number of principal components (PCs) to be kept was chosen based on the Kaiser criterion (SS loadings > 1). Each component was explained using the correlation between axes and variables, and the square cosine value (Cos2) of the variables. The V-test was used to detect whether supplementary qualitative variables were significantly different from 0 [37].

Data on triatomine detection/non-detection in palms were stratified by ecoregion (rain forest/flooded lowlands-savannas), landscape, locality and palm species, with the percentage of palms with at least one captured triatomine and its 95% confidence interval (CI; infestation index) given for each stratum. For comparison within a stratum, conditional maximum-likelihood odds ratios (ORs) with mid-*P* exact 95% CI were calculated, using OpenEpi 3.01. Finally, following the methodology presented in [16], single-season site-occupancy models were used to estimate the prevalence of palm infestation and to investigate ecological correlates of infestation taking into account detection failure [38, 39]. These models were run in the Presence v.2.13.14 software package [40]. The result of each sampling event, i.e. a trap in one palm, was coded 1 if at least one triatomine was captured, and 0 otherwise. As around five traps were placed overnight at the same time for the same palm, this coding provided a history of detection for each palm to build the models (see [16] for more details). Ecological covariates included ecoregion [Amazonian Forest vs flooded lowlands (savanna)], landscape and palm genus.

### Triatomine collection

Triatominae were captured using adhesive bait-traps with mice mostly settled in palms [41], only during one night without repetition. In each capture point, at least five palms were explored for the presence of Triatominae by positioning four to five traps inside the crown of each palm using a 10-m ladder in the late afternoon. Some additional traps were placed, when possible, in holes in the ground. Each trapping site was described, photographed and georeferenced. On the next morning, Triatominae stuck on each trap were collected and the genus and stages of the insect recorded. Adults were transported alive to the laboratory for species determination using dichotomic keys [42, 43] prior to molecular characterization, while the nymphal instars were preserved in 70% alcohol (1 specimen per 1.5-ml tube). The project (field work and use of mice for triatomine capture) was approved by the Bolivian National Bioethics Committee, Department of Research Ethics (CEI-CNB).

### DNA extraction

DNA was extracted to determine the species of triatomine and to identify the blood meal source. For young nymphal-instars (N1–N3), DNA extraction was performed using the total insect. For the oldest nymphal instars (N4, N5) and adults, specimens were first dissected, separating the legs, head and thorax into one tube and the abdomen into another. Equipment used for handling insects was decontaminated with a 10% chlorine solution and rinsed with bi-distilled water before and between each insect dissection. The universal rapid salt-extraction method was applied to all samples [44]. The method is described in detail in Additional file 1: Text S1a.

Due to financial limitations, DNA extraction and analysis were not possible for all specimens. Thus, the processed samples were considered to provide genetic data that were representative coverage of all capture points and positive palms. When fewer than 15 insects were collected at a capture point, DNA was extracted on all the insects to cover the entire available genetic pool. In contrast, when the number of Triatominae collected at a capture point was higher (> 70), only one to two insects per positive palm were processed to cover the greatest genetic variation at that particular capture point. When possible, analysis of the oldest nymphal instars was preferred to that of the youngest.

### Molecular characterization of Triatominae and blood meal source

Characterization of Triatominae was based on the amplification of two fragments of mitochondrial genes, the large ribosomal subunit *16S* (*LSU-rRNA*) and cytochrome B (*Cytb*), and one fragment of a nuclear gene, the *D2* variable region of the *28S* RNA gene (*28S-D2*). The primer sequences used are described in Additional file 1: Table S1 [30, 45, 46], and the methodology is described in Additional file 1: Text S1b. The characterization of the blood meal source was achieved using the set of primers for the *Cytb* fragment (Additional file 1: Table S1) [47], using the methodology described in Additional file 1: Text S1c. Negative control tubes were filled with water and handled in a manner similar to the other tubes containing the insect samples; these were used in each PCR batch.

### Natural infection of Triatominae and discrete typing unit identification

Insect infection by *Trypanosoma* sp. and the discrete typing units (DTUs) of *T. cruzi* were determined by PCR and PCR sequencing of DNA extracted from the whole insect or digestive tract. Two strategies were used. For insects captured during the 2016 mission (Cobija, Guayaramerín and Riberalta), several gene fragments were sequenced: glucose-6-phosphate isomerase (nuclear, *GPI*), gluthathione peroxidase (nuclear, *GPX*), 3-hydroxy-3-methylglutaryl-CoA reductase (nuclear, *HMCOAR*), leucine aminopeptidase (nuclear, *LAP*), pyruvate dehydrogenase E1 component alpha subunit (nuclear, *PDH*), and cytochrome oxidase subunit II (kDNA maxicircle gene, *COII*). The primer sequences used are described in Additional file 1: Table S1 [48, 49] and the methodology is described in Additional file 1: Text S1d.

Insects captured during the 2017 mission were analyzed by mini-exon multiplex PCR (MMPCR) using a mix of primers that enabled *Trypanosoma rangeli* to be distinguished from the three DTU groups of *T. cruzi*: TcI, TcII–TcV–TcVI and TcIII–TcIV [50]. The methodology is described in Additional file 1: Text S1e. The PCR products were distinguished by their molecular weight: 200 bp for TcI, 250 bp for TcII–TcV–TcVI, 150 bp for TcIII–TcIV and 100 bp for *T. rangeli* [47]. As a positive control, DNA of different strains of *Trypanosoma* sp. [*T. rangeli* (RGB), *T. marinkellei* (Tcm) and *T. cruzi*, with the six DTUs TcI (CutiaCl1), TcII (TU18Cl93, CBBCl3), TcIII (M6241Cl6), TcIV (CANIIICl1Z3, 92122102R), TcV (MNCl2, SC43Cl1) and TcVI (TulaCl2, CLBrener)] were used.

Both methods (PCR sequencing and MMPCR) lead to the identification of *T. cruzi* DTUs, but the former gives results about haplotypes while the latter does not. As PCR sequencing is very expensive, MMPCR was used instead of this method for the large collection of bugs in year 2 (2017). Negative control tubes were filled with water and handled in a manner similar to the other tubes containing insect samples; these were used in each batch of PCR.

### Sequence analysis

Direct sequencing of both strands of the amplified PCR products was performed at Macrogen Inc. (Seoul, Korea). The sequences were edited, corrected and aligned using MEGA7 software [51]. Haplotypes were calculated using DnaSP5 software [52]. The BLAST algorithm was used to compare the sequences with those deposited in GenBank (http://www.ncbi.nlm.nih.gov/BLAST). Vector species (*Rhodnius*) were characterized by constructing phylogenetic trees (maximum likelihood) from the obtained sequences and the sequences were deposited in GenBank using IQ-TREE [53]. The best model was determined by ModelFinder [54], and the phylogeny was tested using 1000 ultrafast bootstrap [55]. As the UFBoot values correspond roughly to the probability that a clade is true, a minimum of 95% is considered necessary for a clade to be supported. For each gene fragment, two trees were built: (i) using *Rhodnius* GenBank sequences showing 100% coverage with our haplotypes; (ii) using *Rhodnius* GenBank sequences with less coverage. After alignment of the GenBank sequences, only one randomly selected sequence was retained per haplotype. The second tree (< 100% cover) allowed us to include more species of interest (especially those of *R. pictipes* and *R. prolixus* complexes) and observe how our haplotypes position themselves among the species. A sequence of *T. infestans* was used as an outgroup. Trees were drawn using the R software packages *ggtree* [56] and *treeio* [57]. Haplotype median-joining networks per locus were constructed for *Cytb*, *16S* and *28S-D2* haplotypes with POPART [58, 59].

## Results

### Insect captures

A total of 1200 traps were positioned during the field study, of which 99.4% were located in 238 palms and 0.6% in 32 burrows or ground holes (Table 1). A total of 325 Triatominae were captured, all in palms and all belonging to genus *Rhodnius* (see Additional file 2: Table S2 for the details of each specimen). Across the whole collection of Triatominae, 12% of the traps and 28% of the palms were positive, resulting in 1.6 ± 0.7 insects per positive trap and 3.1 ± 2.7 Triatominae per positive palm. Insects were found at all six locations, with some disparities among them. Most triatomines were found near Trinidad (34% of the insects), San Joaquin (31%) and San Borja (25%). Among the study locations, the positivity of the traps and of the palms ranged from 4% to 19% and from 15% to 43%, respectively (Table 1).

**Table 1:** Collection of insects found in each study locality

Location	No. of specimens	Infestation indices			Colonization index	Crowding indices	
		% Positive traps (positive no./total no.)	% Positive palms (positive no./total no.)	No. in others^a^ (positive no.)	Palms^b^	No. of insects per positive trap	No. of insects per positive palm
*San Borja*							
SB1	0	0% (0/55)	0% (0/11)	0 (–)	–	–	–
SB2	7	6% (6/100)	25% (5/20)	0 (–)	40%	1.2	1.4
SB3	75	43% (38/89)	89% (16/18)	0 (–)	100%	2.0	4.7
Total	82	18% (44/244)	43% (21/49)	0 (–)	86%	1.9	3.9
*San Joaquin*							
SJ1	2	1% (1/75)	7% (1/15)	0 (–)	100%	2.0	2.0
SJ2	0	0% (0/80)	0% (0/19)	0 (–)	–	–	–
SJ3	98	53% (31/59)	83% (10/12)	0 (–)	100%	3.2	9.8
Total	100	15% (32/214)	24% (11/46)	0 (–)	100%	3.1	9.1
*Trinidad*							
T1	105	45% (38/85)	82% (14/17)	3 (0)	100%	2.8	7.5
T2	6	6% (5/84)	18% (3/17)	0 (–)	100%	1.2	2.0
T3	0	0% (0/57)	0% (0/11)	0 (–)	–	–	–
Total	111	19% (43/226)	38% (17/45)	3 (–)	100%	2.6	6.5
*Cobija*							
C1	12	13% (8/60)	36% (4/11)	5 (0)	100%	1.5	3.0
C2	2	4% (1/24)	20% (1/5)	0 (–)	100%	2.0	2.0
C3	3	3% (3/104)	11% (2/18)	14 (0)	100%	1.0	1.5
Total	17	6% (12/184)	21% (7/34)	19 (–)	100%	1.4	2.4
*Guayaramerín*							
G1	3	4% (3/81)	19% (3/16)	0 (–)	100%	1.0	1.0
G2	3	4% (3/71)	14% (2/14)	0 (–)	100%	1.0	1.5
Total	6	4% (6/152)	17% (5/30)	0 (–)	100%	1.0	1.2
*Riberalta*							
R1	2	5% (2/40)	13% (1/8)	0 (–)	100%	1.0	2.0
R2	7	10% (6/61)	33% (4/12)	1 (0)	100%	1.2	1.8
R3	0	0% (0/79)	0% (0/14)	9 (0)	–	–	–
Total	9	4% (8/180)	15% (5/34)	10 (–)	100%	1.1	1.8
Overall total	325	12% (145/1200)	28% (66/238)	32 (0)	95%	2.2	4.9

^a^Others refers to burrow or ground hole

^b^Calculated as: [no. of palms with nymphal instars/no. positive palms] × 100

All nymphal stages were found in the six regions, except in Guayaramerín, where only N1 and N5 were captured (Additional file 1: Table S3). Adults represented only 2.7% of the captures, and were found in only one location in Cobija and in two locations in San Borja. Based on the morphological keys of genus *Rhodnius* [24, 42, 43], in Cobija (location C1; see Table 1 for site identification code ) the one captured specimen, a male, was determined to be *R. robustus*; in San Borja (SB2), the three captured specimens, one male and two females, were determined to be *R. robustus* (they came from three different palms); in San Borja (SB3) one female and one male from the same trap were identified as *R. stali*, and one male and two females from three different palms were identified as *R. robustus*.

### Description of capture points

**Fig. 2:**
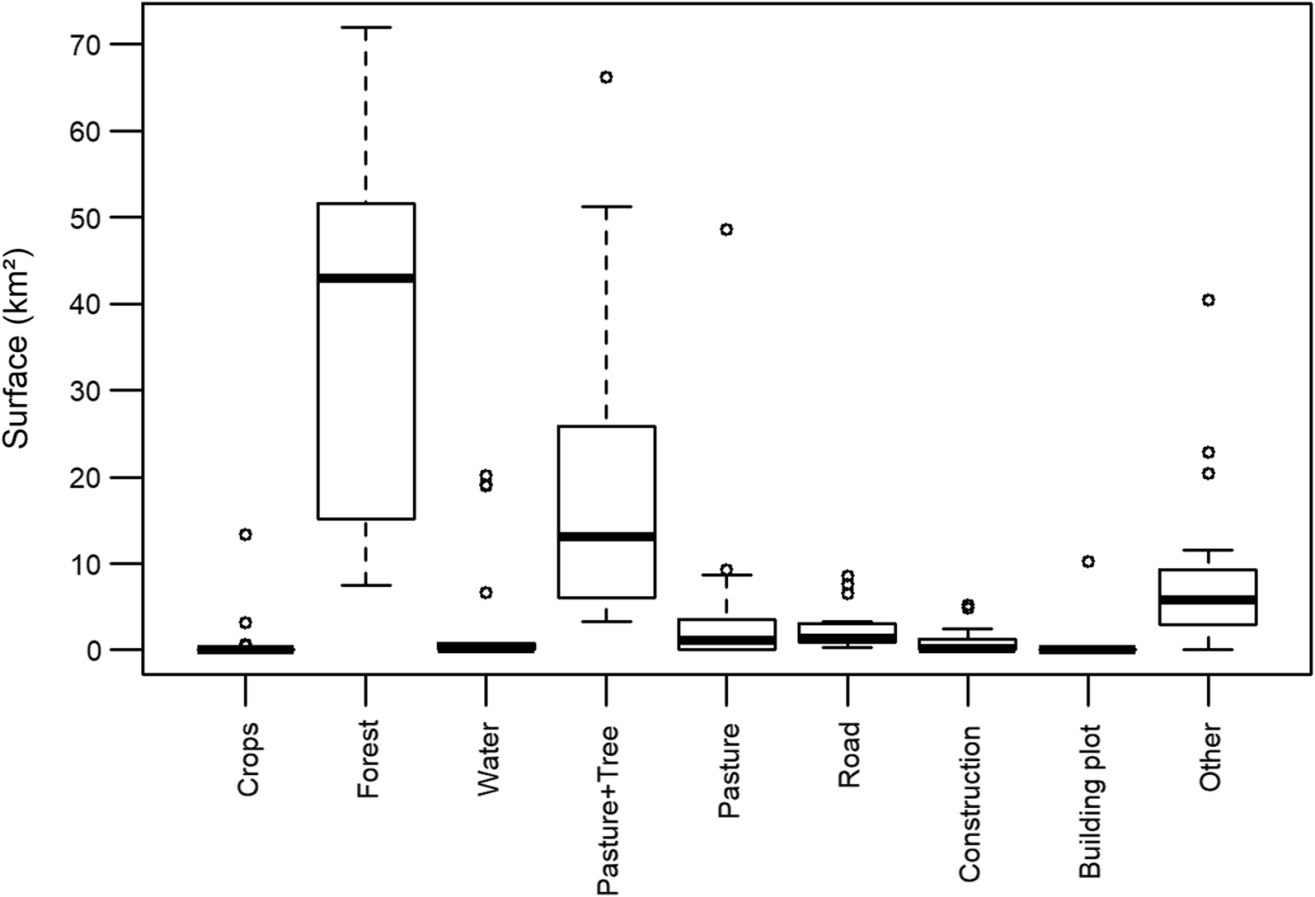
Boxplot of the category environmental surfaces established for each location point. The surfaces (km^2^) are calculated within in a circle of 500 m in radius

**Fig. 3:**
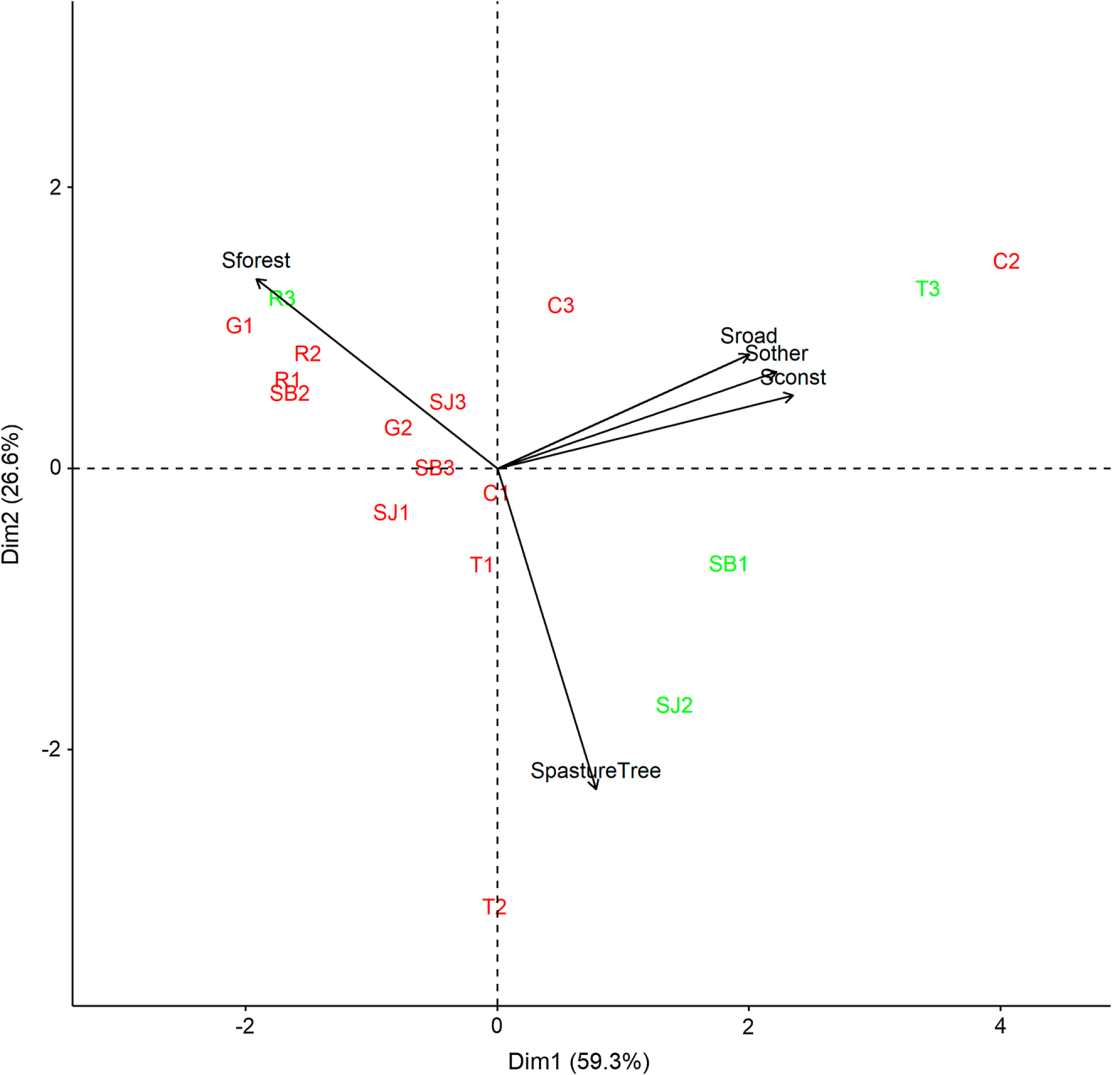
Results of the principal component analysis representing the environment of each capture point. Designations in red are sites where Triatominae were found, otherwise the sites are given in green**.** Variables represent the forest (Sforest), pasture/herbaceous area with isolated trees (SpastureTree), road (Sroad), construction (Sconst) and other (Sother) regrouping peridomiciles, vacant land and land without vegetation. Abbreviations: C, Cobija; G, Guayaramerín; R, Riberalta; SB, San Borja; SJ, San Joaquin; T, Trinidad

The description of the environment around each capture point using a circle of 500 m in radius is shown in detail in Additional file 1: Figure S1 and Additional file 1: Table S4. The environments are best described as forest [mean surface ± standard deviation (SD) of 47% ± 26%] and pasture with isolated trees (24% ± 22%) (Fig. 2). Crops, water, and building plots are poorly represented. The PCA, explaining 85.9% of the variance with two PCs, showed three groups of environments: anthropized area (C2, T3), pasture/herbaceous areas with isolated trees (T2, SJ2) and forested area (R3, G1, R2, R1, SB2) (Fig. 3). The other points fall between these categories. Capture points where no insects were found belonged to all environments (SJ2 is mostly described by pasture, SB1 by both pasture and anthropized area, T3 by anthropized area and R3 by forest). Two qualitative supplementary variables were studied: (i) the rate of insects per trap, and (ii) the rate of positive palms; however, the V-tests were not different from 0, so no relation between these variables and the land use could be established.

**Fig. 4:**
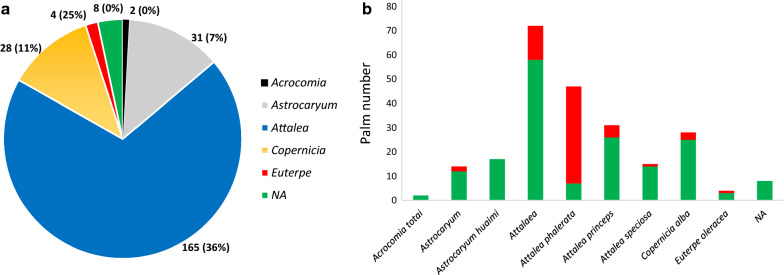
Palm species where triatomines were searched for. **a** The pie chart shows the proportion of each palm genus where trapping was conducted (total number of palms that were surveyed, and percentage of those palms infested with triatomines are given). **b** The barplot shows the number of palms according to the species when known, with red indicating where triatomines were found, otherwise in green). In the case of *Attalea* sp., the bar corresponds to *A. princeps* or *A. phalerata.* *NA* information about genus/species not available

The palms belonged to the following genera: *Attalea* (Kunth) (69%), *Astrocaryum* (G.Mey.) (13%), *Copernicia* (Mart.) (12%), *Euterpe* (Mart.) (2%) and *Acrocomia* (Mart.) (1%) (Fig. 4). The most triatomine-infested genus was *Attalea* (37%). The genus *Euterpe*, with only four trees investigated, also showed infestation in one palm. *Acrocomia* was the only genus where no infestation was found, but only two palms were examined. In terms of palm species, the most infected palm species was *Attalea phalerata* (Mart. ex Spreng.) (85%; Fig. 4), followed by *Euterpe oleracea* (Mart.) (25%), *Attalea princeps* (Mart.) (16%), *Copernicia alba* (Morong) (11%) and *Attalea speciosa* (Mart.) (7%).

**Fig. 5:**
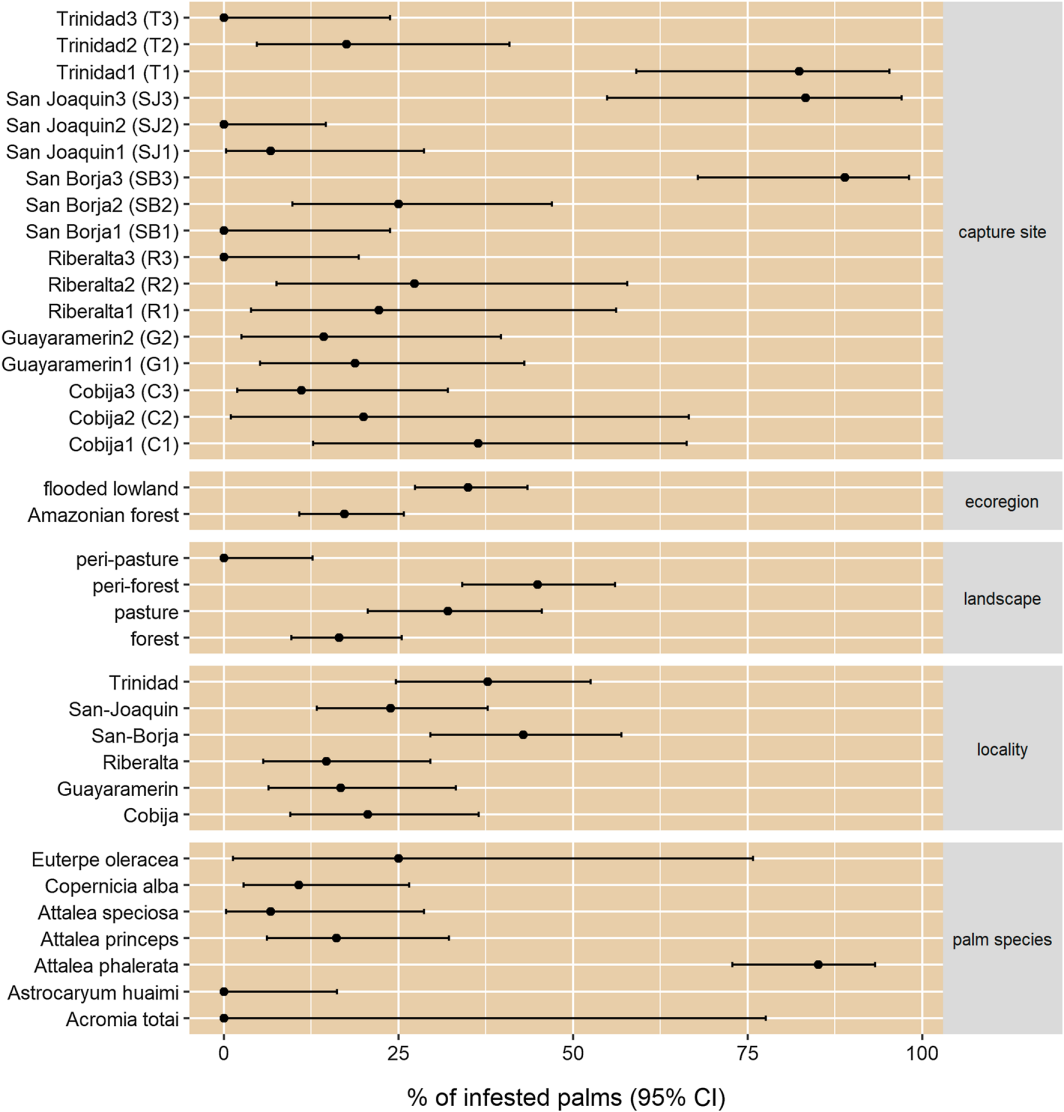
Palm infestation index (95% confidence interval) according to the different strata: capture site, ecoregion, landscape, locality and palm species. A palm was considered infested when at least 1 triatomine was captured. According to ecogregion, Trinidad (T), San Joaquin (SJ) and San Borja (SB) belong to the flooded lowland (savannas) category, and Riberalta (R),  Guayaramerín (G) and Cobija (C) are part of the Amazonian forest category. Landscape was defined by the environment in a circle of 500 m radius around the capture site (see Additional file 1: Table S4; Additional file 1: Figure S1): forest (R1, R2, R3, G1, SJ1, SB2), pasture (T1, T2, SJ2), peri-forest (G2, C1, C2, C3 SJ3, SB3) and peri-pasture (T3, SB1)

Analysis of the infestation index by ecoregions (Fig. 5) revealed that palms are most likely infested in flooded lowlands (savannas) rather than in the Amazonian rainforest (OR: 2.56, 95% CI: 1.37–4.89). The relationship between palm infestation and landscape was also studied (Fig. 5). Of the four landscape categories, namely forest, pasture, peri-forest and peri-pasture (described based on the environment of each capture sites; see Additional file 1: Table S4; Additional file 1: Figure S1), and taking the forest landscape category as the reference category, this analysis showed that palms in the pasture landscape were 2.38-fold more likely to be infested (95% CI: 1.05–5.46), palms in peri-forest were 4.09-fold more likely to be infested (95% CI: 2.00–8.67) and palms in the peri-pasture category were less likely to be infested (OR: 0, 95% CI: 0.00–0.83). In terms of localities (Fig. 5), the percentage of infested palms in Riberalta did not differ from that Cobija (OR: 1.50, 95% CI: 0.41–5.73), Guayaramerín (OR: 1.16, 95% CI: 0.28–4.78) or San Joaquin (OR: 1.81, 95% CI: 0.57–6.38). However, palms from Trinidad were 3.47-fold (95% CI: 1.16–11.79) more likely to be infested than those from Riberalta, and the OR increased to 4.28 (95% CI: 1.46–14.3) in San Borja. Finally, among palm species, *A. phalerata* was more likely to be infested than the others (OR: 71.63, 95% CI: 10.47–1739 when *A. speciosa* was used as reference, or OR: 27.76, 95% CI: 8.39–107.4 when *A. princeps* was used as reference).

The palm infestation index calculated over all sites was 0.121 (145 positive traps/1200 surveyed traps). A null single-season site-occupancy model that considered a constant occupancy and a constant rate of detection, estimated an overall palm occupancy rate of 0.306 (95% CI: 0.246–0.467), which is 39.5% higher than the observed index. Estimates of palm occupancy by ecoregion showed that the odds of occupancy are 2.79-fold higher at a savanna site than at a forest site (95% CI: 1.43–5.41). The palm occupancy estimate for savanna sites was 0.395 (95% CI: 0.309–0.468), which is 44% higher than the observed index (0.174); in comparison, it was only 26% higher for the Amazonian forest (estimate palm occupancy: 0.189, 95% CI: 0.120–0.285). The models on the influence of landscape showed that compared to forest sites, the odds of occupancy at sites in peri-forest (border between human settlement and forest) were 2.91-fold higher (95% CI: 1.34–6.29) and the odds of occupancy at sites in pasture were 2.53-fold higher (95% CI: 1.03–6.24). Finally, the model on palm genus, and using *Copernicia* as reference, showed only one significant result where the odds of occupancy of *Attalea* were 6.49-fold higher (95% CI: 1.84–22.85).

### Molecular characterization of triatomines

DNA was extracted from 114 insects (35% of the total number of captured insects; see DNA extraction section in Methods section) for which at least one gene fragment (*Cytb*, *16S*, or *28S-D2*) was sequenced. From the 110 *Cytb* samples sent for sequencing, 100 sequences were obtained, of which 99 were based on both forward and reverse directions, and one was obtained from the forward direction only. The details of each sequence, and the BLAST result for each, are presented in Additional file 2: Table S2. Two sequences were removed from the haplotype analyses, one being too short compared to the others (only 465 bp), and one having two polymorphic loci (C/T). Hence, based on the 98 aligned sequences (607 bp), 17 haplotypes were detected: 14 corresponding to *R. robustus/R. prolixus* (100% cover, 99.34–100% identity) and three belonging to *R. stali* (96–100% cover, 98.68–100% identity) as putative species, with 81 and 17 individuals, respectively (Additional file 1: Table S5). For the *16S* fragment, 87 insects were analyzed and 72 sequences were obtained using both strands, except for one sequence based only on the reverse sequence. Eight haplotypes of 431 bp were detected, four corresponding to *R. prolixus* (99–100% cover, 98.83–99.70% identity) or *R. robustus* (69–78% cover, 98.82–100% identity) and four corresponding to *R. stali* (100% cover, 98.60–100% identity); the number of involved specimens was 59 and 13, respectively (Additional file 1: Table S5). For the *28S-D2* fragment gene, 29 individuals were analyzed, and 24 sequences were obtained (578 bp long after alignment), all from both strands except for one based on the forward sequence. Two sequences showing two polymorphic loci (T/C) were deleted before haplotype analysis. Four haplotypes were detected, all corresponding to the putative species *R. robustus* (100% cover, 99.48–100% identity, Additional file 1: Table S5). A total of 86 insects were studied by two gene fragments (74 *R. robustus/R. prolixus*, 12 *R. stali*), and 23 insects were studied by only a single gene fragment (17 *R. robustus/R. prolixus*: 3 based on *16S*, 8 on *Cytb* and 6 on *28S-D2*; and 6 *R. stali*: 1 based on *16S* and 5 on *Cytb*;Additional file 2: Table S2). All sequences (*Cytb*, *16S*, *28S-D2*) described in the present study have been deposited in GenBank (accession numbers are given in Additional file 2: Table S2).

**Fig. 6:**
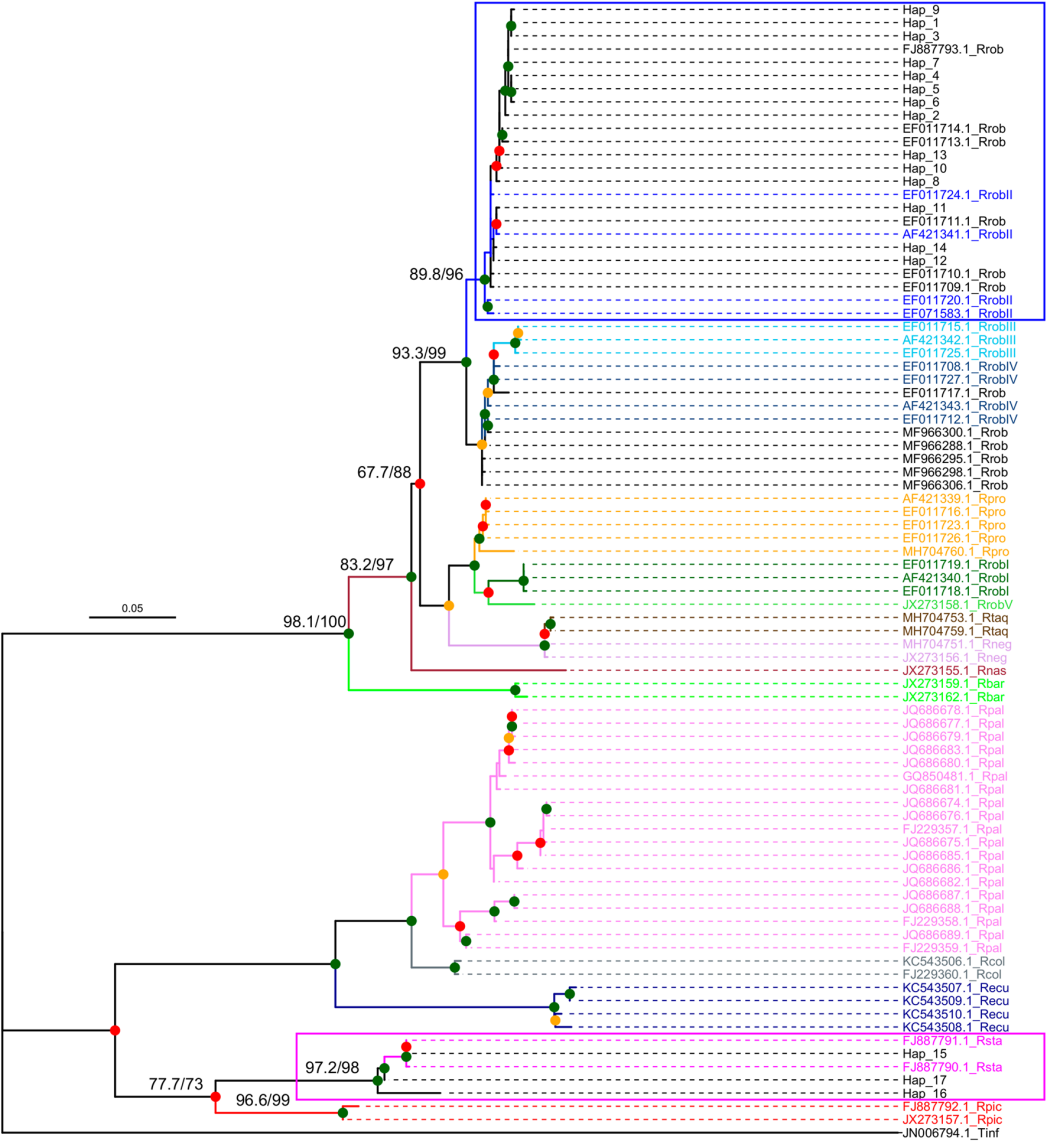
Maximum likelihood tree constructed with the 17 current cytochrome B (*Cytb*) haplotypes jointly with 100%-coverage sequences deposited in GenBank: 69 individuals belonging to 11 species (608-bp alignment). Tree was constructed using IQ-Tree after determining the best substitution model using ModelFinder. UFBoot values based on 1000 repetitions are shown on the nodes by colors (green: > 95%, orange: 90–95%, red: 70–90%). SH-aLRT support (%)/ultrafast bootstrap support UFBoot (%) are shown for the principal nodes of interest. Species and subclades of *R. robustus* sensu lato are differentiated by colors. *Triatoma infestans* sequence was used as the outgroup (Tinf). FJ887793.1_Rrob is identical to Hap_7, and FJ887791.1_Rsta is identical to Hap_15. Species abbreviations: Rbar, *R. barretti*; Rcol, *R. colombiensis;* Recu, *R. ecuadoriensis*; Rnas, *R. nasutus*; Rneg, *R. neglectus*; Rpal, *R. pallescens*; Rpic, *R. pictipes*; Rpro, *R. prolixus*; Rrob, *R. robustus* and its subclades (RrobI–RrobV); Rsta, *R. stali*; Rtaq, *R. taquarussuensis*.

According to the above results, the putative current species belong to two complexes: the *R. pictipes* complex, and the *R. prolixus*–*R. robustus* cryptic species complex. A first phylogenic analysis of *Cytb* sequences with those of genus *Rhodnius* from GenBank (608-bp alignment) included two sequences of *R. barretti* (Abad-Franch et al., 2013), two of *R. colombiensis* (Moreno Mejía, Galvão & Jurberg, 1999), four of *R. ecuadoriensis* (Lent & León, 1958), one of *R. nasutus* (Stål, 1859), two of *R. neglectus*, 19 of *R. pallescens* (Barber, 1932), two of *R. pictipes*, five of *R. prolixus*, 27 of *R. robustus*, of which 15 were identified by the 5 subclades (I to V) described by [29, 30] and 12 sequences were without this identification, two of *R. stali* and two of *R. taquarussuensis*. The GenBank accession numbers of the sequences are given in Fig. 6. Phylogenic analyses show that *Cytb* haplotypes (hap) 1–14 are closely related to *R. robustus* sequences; more precisely, they are clustered with the *R. robustus* II subgroup with a support of 96% UFboot, with this group (Bolivian haplotypes 1–14 and *R. robustus* II) being separate from the other members of the complex. Haplotypes 15–17 are closely related to *R. stali* sequences with a support of 98% UFboot, apart from *R. pictipes* (Fig. 6). These results confirm the putative species identified by the BLAST analysis. Using shorter sequences (369 bp) it was possible to include *R. brethesi* and *R. montenegrensis* species in the *Cytb* analysis (Additional file 1: Figure S2). In this case, haplotypes 1–14 are still grouped in the *R. robustus* II clade with 98% UFBoot support, along with the *R. montenegrensis* sequence. It is worth noting that due to the use of a shorter sequence length (369 bp), the species of the *R. pictipes* complex are no longer well discriminated, i.e. KC249235.1 *R. brethesi* became identical to KC249236.1 *R. pictipes* (Additional file 1: Figure S2).

Phylogenic analyses of the *16S* gene fragment using 100% coverage resulted in the inclusion of nine GenBank sequences belonging to eight *Rhodnius* species (Additional file 1: Figure S3). Hap1 to hap4 were included in a clade comprising *R. domesticus*, *R. neglectus*, *R. neivai* and *R. prolixus*, with 99% UFBoot support, but without separation between species, well separated from hap5 to hap8 that clustered with *R. stali*, *R. brethesi* and *R. pictipes*. The tree using the sequences with < 100% coverage allowed the inclusion of more species, especially *R. robustus* (Additional file 1: Figure S3). Hap1 to hap4 were included in a clade with *R. robustus*, *R. neglectus*, *R. prolixus*, *R. domesticus* and *R. neivai w*ith 96% UFboot support. Hap5 to hap8 were separate from the *R. pictipes* clade (96% UFboot), but they were not distinguishable between *R. stali* and *R. brethesi*.

For the *28S*-*D2* fragment, only six sequences belonging to three species of genus *Rhodnius* with 100% coverage with our haplotypes (578 bp) were available in GenBank: *R. robustus, R. prolixus* and *R. nasutus*; no species conclusion can be extracted from the phylogenetic tree (Additional file 1: Figure S4). Using shorter sequences, 21 GenBank sequences belonging to nine species were included in the phylogenic tree. The four haplotypes were classified in a clade (96% UFBoot) comprising *R. colombiensis*, *R. ecuadoriensis*, *R. nasutus*, *R. neglectus*, *R. pallescens*, *R. prolixus* and *R. robustus*, separated from *R. stali* and *R. pictipes*.

**Fig. 7:**
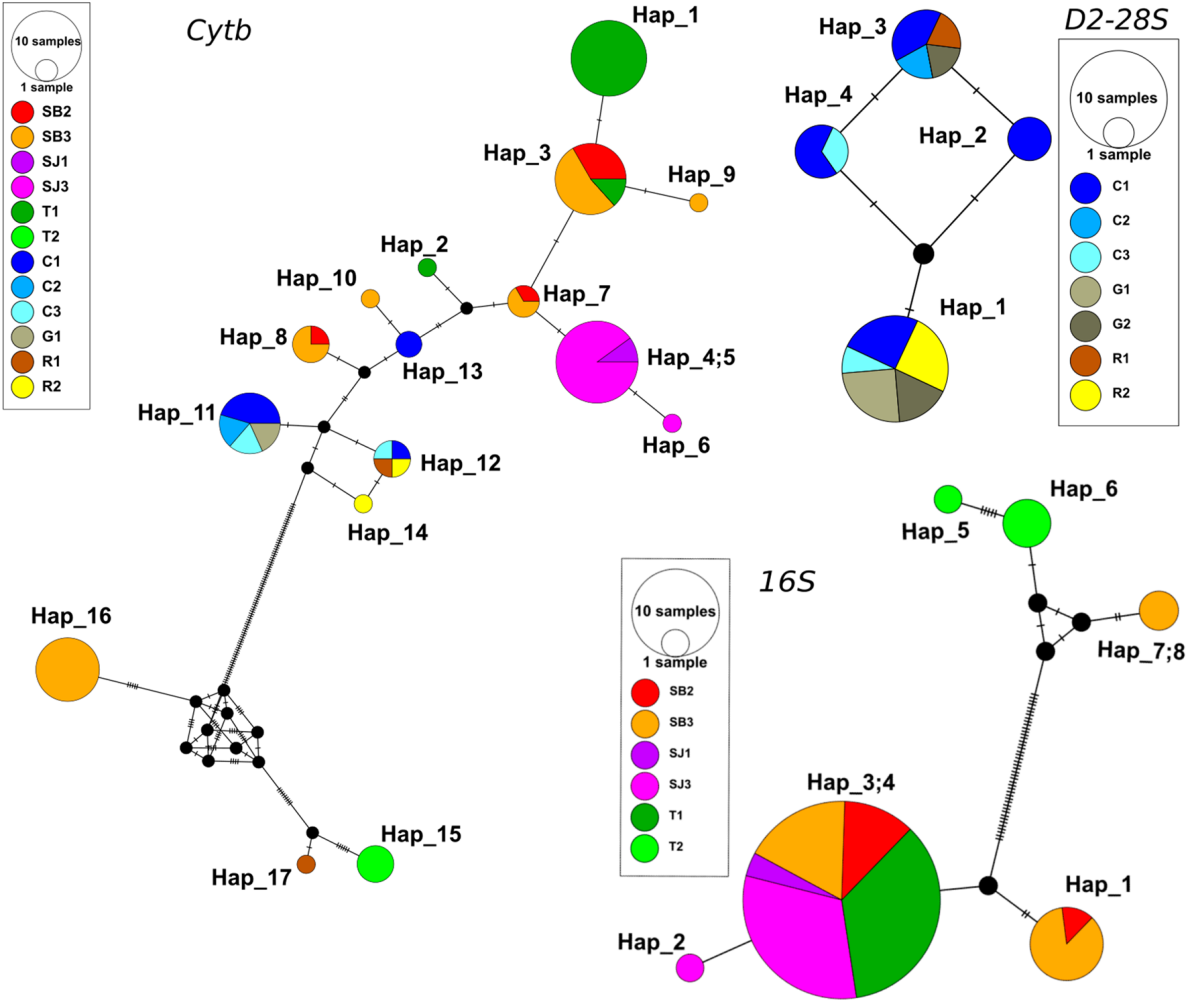
Haplotype networks for *Cytb*, *16S* and *28S-D2* sequence fragments. The colors represent the geographical origin. For *Cytb*, hap1 to hap14 correspond to *R. robustus*, and hap15 to hap17 correspond to *R. stali*. For *16S*, hap1-4 corresponds to *R. robustus*, and Hap5 to hap8 correspond to *R. stali*. For *28S-D2*, all haplotypes correspond to *R. robustus*. Each tick on the branches represents a mutational step. Abbreviations: 16S, large ribosomal subunit* 16S*; D2-28S, *D2* variable region of the *28S* RNA gene, Cytb, Cytochrome B

Our results appear to show some geographic structuring of haplotypes after construction of the network, especially for *Cytb* (Fig. 7; Table 2). For *R. robustus*, this involves hap1 and hap2, which were found only in Trinidad; hap4, hap5 and hap6, in San Joaquin; hap7, hap8, hap9 and hap10 in San Borja; hap13 in Cobija; and hap14 in Riberalta. The remaining haplotypes were shared in different regions: hap3 was shared between San Borja and Trinidad; hap11, between Cobija and Guayaramerín; and hap12, between Cobija and Riberalta. San Borja and Cobija showed the greatest haplotype diversity for *Cytb* (Table 2). For *R. stali*, each of the three haplotypes was found in only one region: hap15 in Trinidad, hap16 in San Borja and hap17 in Riberalta. For the *16S* characterizing *R. robustus*, hap1 was found only in San Borja, hap2 only in San Joaquin and hap3 and hap4 in the three regions of San Borja, San Joaquin and Trinidad. For the *16S* characterizing *R. stali*, hap5 and hap6 were only found in Trinidad, and hap7 and hap8 only in San Borja. Regarding *28S-D2*, all four haplotypes were found in Cobija, while in Riberalta and in Guayaramerín, only hap1 and hap3 were found. Haplotype diversity tended to be higher in San Borja, with six haplotypes using 34 sequences of* Cytb* [Hd = 0.727 ± 0.047; 1 haplotype corresponding to *R. stali* (12 sequences) and 5 haplotypes corresponding to *R. montenegrensis (*22 sequences, Hd = 0.623 ± 0.099)] and Riberalta, with three haplotypes using four sequences of* Cytb* [Hd = 0.833 ± 0.222; 1 haplotype corresponding to *R. stali* and 2 haplotypes corresponding to *R. montenegrensis *(3 sequences; Hd = 0.667 ± 0.314)], then Cobija, with three haplotypes, 13 sequences [Hd = 0.513 ± 0.144 (all of them corresponding *R. montenegrensis*)] and Trinidad, with four haplotypes, 24 sequences [Hd = 0.482 ± 0.111 (1 corresponding to *R. stali* and 3 to *R. montenegrensis*: Hd = 0.279 ± 0.123).

**Table 2:** *Cytb*, *16S* and *28S-D2* haplotypes and corresponding species according to the BLAST procedure and phylogenic analyses together, and geographic distribution

Capture point^a^	*Cytb* Haplotype			*16S* Haplotype			*28S-D2* Haplotype		
	Name^b^	Putative species^c^	No. specimens	Name^b^	Putative species^c^	No. specimens	Name^b^	Putative species^c^	No. specimens
*San Borja*									
SB2	Hap3-*Cytb* Hap7-*Cytb* Hap8-*Cytb*	*R. r* *R. r* *R. r*	5 1 1	Hap1-*16S* Hap3-*16S*	*R. p./R. r* *R. p./R. r*	1 6	–		
SB3	Hap3-*Cytb* Hap7*-Cytb* Hap8-*Cytb* Hap9-*Cytb* Hap10-*Cytb* Hap16-*Cytb*	*R. r* *R. r* *R. r* *R. r* *R. r* *R. s*	8 2 3 1 1 12	Hap1-*16S* Hap3-*16S* Hap4-*16S* Hap7-*16S* Hap8-*16S*	*R. p./R. r* *R. p./R. r* *R. p./R. r* *R. s* *R. s*	6 7 2 1 8	–		
*San Joaquin*									
SJ1	Hap4-*Cytb* Hap5-*Cytb*	*R. r* *R. r*	1 1	Hap3-*16S*	*R. p./R. r*	2	–		
SJ3	Hap4-*Cytb* Hap6-*Cytb*	*R. r* *R. r*	18 1	Hap2-*16S* Hap3-*16S*	*R. p./R. r* *R. p./R. r*	1 16	–		
*Trinidad*									
T1	Hap1-*Cytb* Hap2-*Cytb* Hap3-*Cytb*	*R. r* *R. r* *R. r*	17 1 2	Hap3-*16S*	*R. p./R. r*	18	–		
T2	Hap15-*Cytb*	*R. s*	4	Hap5-*16S* Hap6-*16S*	*R. s* *R. s*	1 3	–		
*Cobija*									
C1	Hap11-*Cytb* Hap12-*Cytb* Hap13-*Cytb*	*R. r* *R. r* *R. r*	5 1 2	–			Hap1-*D2* Hap2-*D2* Hap3-*D2* Hap4-*D2*	R. p. complex R. p. complex R. p. complex R. p. complex	3 2 2 2
C2	Hap11-*Cytb*	*R. r*	2	–			Hap4-*D2*	R. p. complex	1
C3	Hap11-*Cytb* Hap12-*Cytb*	*R. r* *R. r*	2 1	–			Hap1-*D2* Hap3-*D2*	R. p. complex R. p. complex	1 1
*Guayaramerín*									
G1	Hap11-*Cytb*	*R. r*	2	–			Hap1-*D2*	R. p. complex	3
G2	–			–			Hap1-*D2* Hap4-*D2*	R. p. complex R. p. complex	2 1
Riberalta									
R1	Hap12-*Cytb* Hap17-*Cytb*	*R. r* *R. s*	1 1	–			Hap4-*D2*	R. p. complex	1
R2	Hap12-*Cytb* Hap14-*Cytb*	*R. r* *R. r*	1 1	–			Hap1-*D2*	R. p. complex	3

*16S* Large ribosomal subunit *16S*,* Cytb* cytochrome B, *28S-D2* *D2* variable region of the *28S* RNA gene

^a^See Table 1 for collection site code

^b^All haplotypes were identified based on sequencing of both strands, with exception of Hap5-Cytb (only forward strand) and Hap5-16S (only reverse strand)

^c^*R.r*,* R. robustus*;* R. p*,* R. prolixus*;* R. p/R. r*, one of the two species;* R. p *complex,, *R. prolixus* complex of species; *R. s.*,* R. stali*

### Blood meal source

A total of 63 blood meal sources could be determined from 68 DNA extractions, with all sequences based on the two strands (Additional file 2: Table S2). These were determined from DNA extracted from 61 nymphal instars (2 N2, 32 N3, 19 N4 and 8 N5) and two adults (1 male and  1 female), from collection sites at San Borja, San Joaquin and Trinidad. Seven blood meals were attributed to *Mus musculus* (Linnaeus, 1758) and were likely due to insects that fed on the mouse-bait used for trapping. Four other blood meals were detected from two species of Didelphidae (Didelphinae subfamily), all in San Borja (SB3): two blood meals on *Philander canus* (Osgood, 1913), from two nymphs captured in two different palms, and two blood meals on *Metachirus nudicaudatus* (Geoffroy, 1803), from one N3 and one male captured with the same trap. Both species of Didelphidae are reported in Beni Department, Bolivia. Three blood meals were attributed to rodents, all from nymphs from Trinidad (T1): two on *Oecomys mamorae* (Thomas, 1906) (Cricetidae family, Sigmodontinae subfamily), a widespread species in the country, from two insects captured in two different palms; and one blood meal on *Coendou prehensilis* (Linnaeus, 1758) or *Coendou bicolor* (Tschudi, 1844) (Erethizontidae family), both reported from the region. One blood meal was attributed to Reptilia species: *Hemidactylus mabouia* (Moreau de Jonnès, 1818) (Gekkonidae family), found in a nymph from San Joaquin (SJ3). This species, native to Africa, has been introduced by humans and is widely reported in South America. Finally, 48 blood meals had a human origin; these were found in San Borja [in SB2 (1 female) and in SB3 (11 nymphs in 7 palms], in San Joaquin (SJ3; 19 nymphs in 5 palms) and in Trinidad (T1; 17 nymphs in 7 palms). According to the description of the capture points (Additional file 1: Figure S1; Additional file 1: Table S4), SB2 is located in a forest area, with the nearest house 41 m away, SB3 is located in an indigenous village, with the shortest distance to a house being 9 m, SJ3 is a brickyard, with the smallest distance to a house being 9 m, and T1 was in a forest fragment at the border of a pasture, in front of a ranch (58 m). All individuals that had fed on human blood were found in *A. phalerata* (Additional file 2: Table S2). It is worth noting that the negative controls used in each batch of PCR never amplified any DNA.

### Infection

**Fig. 8:**
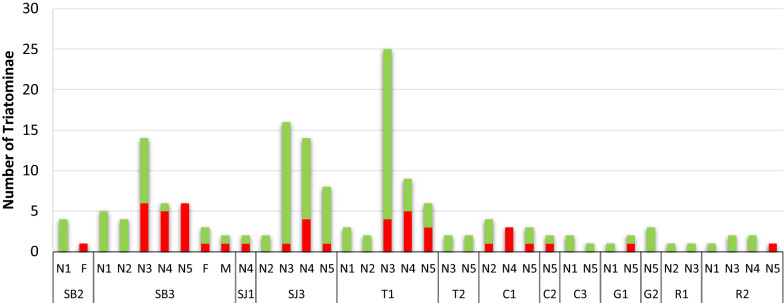
Triatominae analyzed for *Trypanosoma* infection at each capture point, stage (N1–N5), sex (F, M), and infection rate (positive in red, negative in green). All were infected by the DTU TcI, except for two N3 in capture point T1 that were infected by *T. rangeli*, and 3 N5 and a female in capture point SB3 that had a mixed infection TcI-*T. rangeli*. For Cobija, Guayaramerín and Riberalta, insects returned from sequencing with bad sequences were considered in this figure to be negative. Abbreviations: DTU, Discrete typing unit; N1–N5, nymphal instars 1–5; M, male; F, female; for abbreviations of capture points, see Fig. 3 caption

A total of 165 insects were analyzed for detection and identification of *Trypanosoma* sp., of which 136 were analyzed using MMPCR and 29 were analyzed by sequencing of gene fragments. From these, 48 samples were found to be positive (Additional file 2: Table S2), infected by *T. cruzi* (42 insects, 87.5%), *T. rangeli* (2 insects, 4.2%) and a mixed infection *T. cruzi*–*T. rangeli* (4 insects, 8.3%), as shown after agarose gel electrophoresis (Additional file 1: Figure S5). All *T. cruzi* belonged to the TcI strain (Additional file 2: Table S2). The infection rate by locality was 44.4% (20 infected/45 insects) in San Borja, 24.5% (12/49) in Trinidad and 16.7% (7/42) in San Joaquin, with a difference observed between capture points within a locality (Fig. 8). *Trypanosoma rangeli* was found in Trinidad and in San Borja. According to the putative species, 35% (22/62) of the *R. robustus* and 21% (3/14) of the *R. stali* were found infected by *Trypanosoma* sp. *Trypanosoma rangeli* was found in one *R. robustus* (mixed infection).

Of the 32 insects captured in Cobija, Guayaramerín and Riberalta, 29 were studied for *Trypanosoma* infection by sequencing several gene fragments from positive and negative PCR products: six fragments in four insects and two fragments (*GPI* and *GPX*) in 25 insects were sequenced. Readable sequences were obtained for only nine insects, with all readable sequences corresponding to the TcI DTU (Additional file 2: Table S2). The number of infected insects per locality was seven in Cobija (2 N2, 3 N4, 2 N5), one N5 in Guayaramerín and one N5 in Riberalta (Fig. 8). Seven infected insects were determined to be *R. robustus*; the other two insects were unidentified.

## Discussion

### Triatomine abundance

Knowledge about the vectors of Chagas disease in the Bolivian Amazon region is scarce. This study, with its wide geographical range, highlights the ubiquity of *Rhodnius* in the region, which is an important novelty. Indeed, these insects were captured in each of the six study localities. Insect abundance (> 80 vs < 17 insects) and trap positivity (> 15% vs < 6%) were higher in the three flooded lowland (savanna) capture points than in the three Amazonian forest ones surveyed 1 year before. Palm occupancy models showed that palms in flooded lowlands are 2.8-fold more likely to be infested than palms in the Amazonian forest. Our estimates are in the range of previous estimates found for central (24%) and southwestern Amazonia (37%) [16]. The landscape is also important. According to our results, palms at the border between human settlement and forest are 2.9-fold more likely to be infested than palms in forest, and that palms in a pasture environment are 2.5-fold more likely to be infested than palms in the forest. Although all collections were conducted during the dry season (August) and with the same methodology, local variations (climate, wildlife, etc.) between years may explain these differences between savanna and Amazonian forest. More specifically, these regions are under the influence of global climate variations, such as El Niño and La Niña, which can result in drought or flood years. Precipitation data collected from the SENAMHI (National Service of Meteorology and Hydrology, Bolivia; http://senamhi.gob.bo) at each collection point except Guayaramerín (for which no data were available) showed that the total amount of precipitation during the year preceding the field missions was higher for San Borja (4265 mm), similar for San Joaquin (3308 mm) and Riberalta (3300 mm) and lower for Cobija (2986 mm) and Trinidad (2975 mm). Thus, the total amount of precipitation during this previous year does not seem to explain the differences observed between the flooded lowlands and the Amazonian forest ecoregions, although the importance of monthly variation cannot be discarded. Further studies with a specific design should be carried out to truly assess the abundance and stability of *Rhodnius* populations over time, study the possible factors related to annual variation and confirm the present results. It is important to note that very few adults (2.7%) were found, while all the nymphal instars were well represented. The density (no. of *Rhodnius*/palm: 0.13–8.16) and crowding (no. of* Rhodnius*/positive palm: 1.00–9.80) indices observed in this study are similar to those obtained in a previous study in Bolivia conducted in Yucumo, 50 km southwest of San Borja (density: 0.06–1.46; crowding: 1.33–6.33 [21]). They are also similar to those obtained in a study of *R. ecuadoriensis* in Ecuador (density: 0.06–2.55, crowding: 1.00–10.21 [17]).

### Triatomine species and geographic distribution

In Bolivia, four species of *Rhodnius* have been described to date: *R. robustus*, *R. stali*, *R. pictipes* and newly described *R. micki* [24]. Our results using the *Cytb* fragment demonstrate that hap1 to hapl14 are clustered with clade II of *R. robustus*, now named *R. montenegrensis* [27]. *Cytb* hap15 to hap17 were identified as *R. stali* species, with a high Uboot support value of 98%. *Rhodnius micki*, a newly described species from the *R. pictipes* complex, was not included in this analysis due to the lack of sequences available in GenBank. Nevertheless, the adults captured in this study were morphologically consistent with *R. stali* and not with the description of *R. micki* [24]. In conclusion, all analyzed insects belong to two species: *R. montenegrensis* and *R. stali*. This makes the existence and true distribution of *R. robustus* sensu stricto in Bolivia doubtful, and more sampling and genetic analysis are essential to resolve this important issue.

*Rhodnius montenegrensis* was found in all six geographic localities, whereas *R. stali* was only captured in San Borja, Trinidad and Riberalta. The number of specimens was also higher for *R. montenegrensis*. Interestingly, *R. stali* was predominant in a previous study of *Rhodnius* in palms in Yucumo, 50 km southwest of San Borja (Andean foothills, October 2012), where the authors identified as putative species 23 *R. stali* and one *R. robustus* [21]. In the Alto Ben region of La Paz Department, 65 km south of Yucumo, *R. stali* was the only *Rhodnius* species found in palms (*Attalea phalerata)* [11]; this species was very abundant and in a peridomicile domiciliation process in the same region in 2006 [10]. This panorama allows us to speculate that *R. stali*, although widely distributed in the lowlands of the Amazon region of Bolivia, could be more abundant at the foot of the Andes. *Rhodnius robustus* (identified only by morphology, so potentially *R. montenegrensis*) was also captured in the municipality of Villa Tunari of the department of Cochabamba [22]. Therefore, it would appear that *R. montenegrensis* has an extensive home range in Bolivia. For both species, with the majority of haplotypes observed in only one location, a geographic structuring of haplotypes seems to exist, which was especially highlighted with *Cytb*. Haplotype diversity tended to be highest in San Borja, which is the closest point to the Andean foothills and an entry point into the Amazonian region for the human population. Interestingly, the four sequences collected in Riberalta corresponded to three haplotypes, also suggesting high diversity in the Amazonian forest. Further research on insect collection and haplotype diversity should be conducted in the future to investigate diversity in these regions.

### Infection of Triatominae by *Trypanosoma*

Both species were detected naturally infected with *Trypanosoma* sp., with an infection rate of 35% for *R. montenegrensis* and 21% for *R. stali*. Most infections were due to *T. cruzi*, specifically for the DTU TcI. A variation in infection rate was observed between localities, being higher in San Borja (44.4%) and lower in San Joaquin (16.7%), for a similar number of Triatominae tested (45 vs 42, respectively). Six specimens were found to be infected with *T. rangeli* (4 individuals with mixed infection *T. rangeli*-TcI). This is interesting because *T. rangeli* is known to occur in primates (Squirrel Monkey) in Bolivia [60], but this is the first time this species has been shown in Triatominae. *Trypanosoma rangeli* is a well-known parasite that is restricted to Central and South America, of which Bolivia is the southern border. It can infect a wide range of mammals, including humans (not pathogenic in humans), and is mainly transmitted by *Rhodnius* species. In species such as *R. ecuadoriensis* [61] or *R. pallescens* [62], infection with *T. rangeli* and mixed *T. rangeli/T. cruzi* infection are common occurrences. Our results allowed us to identify one of the species infected by this parasite, namely, *R. montenegrensis*. Infection of this species with *T. rangeli* has been previously observed in Brazil [63]. *Rhodnius stali* has also recently been found to be infected with *T. rangeli* in Brazil [64]. This is a very important discovery because there is a tendency to believe that *T. rangeli* is not present in Bolivia. The two species are not easily distinguishable by microscopy, and cross-reactions occur with serological tests, causing false-positive results [65]. This is a problem that the Ministry of Health and the Chagas Program in Bolivia should take into account.

### Palm species and their infestation

Palms are an emblematic group with > 2500 species distributed throughout the world [66]. In the Amazon rainforest, palms represent 60% of the most common species [67]. They are key species for human populations, which use their leaves for the construction of roofs, their fruits for food and handicraft, among others [68]. It is customary for local people to keep some palms near their dwellings to provide shade and fruits for domestic animals. These palms are also important for wildlife, providing shelter and food [66]. Palms have been shown to provide Triatominae with a refuge characterized by stable abiotic conditions [69]. Local wildlife in association with palms is the food source that Triatominae needs to thrive [69]. *Rhodnius* species have been found in close association with palms, with some species being captured exclusively in one palm species, such as *R. brethesi* and *Leopoldinia piassaba* (Wallace), while others live in various palm species [69]. In our study, *R. stali* was found in two palm species, i.e. *Copernicia alba* and *Attalea phalerata*, while *R. montenegrensis* was captured in *A. phalerata*, *A. speciosa* and *A. princeps*. Other non-identified *Rhodnius* specimens were also found in *Astrocaryum* sp. and *Euterpe oleracea*. *Copernicia alba* is a synonym of *C. australis*, where *R. pictipes* has been found previously [70]. Our study is the first to report the presence of *R. stali* in a palm species other than *A. phalerata* [69], although it is possible that this reported *R. pictipes* in *R. alba* was in fact *R. stali*. *Rhodnius montenegrensis* has been found previously in *A. speciosa* [63, 71], and *R. robustus* has previously been found in various palms, including *Astrocaryum aculeatum* (G.Mey.)*, Astrocaryum murumuru* (Mart.), *Attalea phalerata* and *A. speciosa*. Here, we report two new species for *R. montenegrensis*: *Attalea princeps* and *A. phalerata*; these two palms are widely distributed in Bolivia and are known locally as Motacú. Finally, Acai (*E. oleracea*) is known to be infested by Triatominae [*Alberprosenia malheiroi* (Serra, Atzingen & Serra, 1987)] [16]. Unfortunately, we were unable to determine which species inhabited these palms in Bolivia. It is interesting to note that both *Rhodnius* species were found twice sharing the same palm (*A. phalerata*), in San Borja (SB3): two N1 nymphs in one case (same trap) and one N2 (*R. montenegresensis*) and one N4 (*R. stali*) in two different traps in the other case. The crown structure and abundance of vegetable matter and epiphytes were unfortunately not described in our study. They are factors known to increase the probability of infestation and the size of the triatomine colonies in palm [17, 72]. We have captured *Rhodnius* specimens in species such as *Astrocaryum* sp. and *C. alba* that have less developed crowns than *Attalea* sp. Therefore, it would be interesting to investigate this point in future work.

### Blood source meals

The Amazon forest is the biome with the largest number of mammals infected with *T. cruzi* in Brazil [73]. Blood meal analysis provides an overview of the sylvatic hosts of *Rhodnius* in Bolivia: Didelphidae (*Philander canus* and *Metachirus nudicaudatus*), rodents (*Omamorae mamorae*, and *Coendou prehensilis/C. bicolor*) and Reptilia (*Hemidactylus mabouia*). *Oecomys concolor* (Wagner, 1845) [74] and *Oecomys trinitatis* (Allen & Chapman, 1893) [75] have been previously cited as hosts of *R. prolixus*, but our study is the first to report the detection of *O. mamorae*, a rodent from Bolivia, as host, having been detected with *R. montenegrensis*. *Coendou* (*prehensilis*, *bicolor*, or sp.) is a known host of *R. pallescens*, *R. prolixus* and *Panstrongylus rufotuberculatus* (Champion, 1899), and an association has been reported for *Eratyrus mucronatus*, *Panstrongylus geniculatus*, *Panstrongylus lignarius* (Walker, 1873) and *Triatoma dimidiata* (Latreille, 1811) [74, 75]. Opossums are well-known hosts of Triatominae and have been reported for many species. Nevertheless, *P. canus* has never been reported as a host, and *M. nudicaudatus* has only been reported as a host in *Rhodnius pallescens* (Barber, 1932) [74] and *R. prolixus* [75]. These two hosts were detected with *R. montenegrensis*. *Hemidactylus mabouia* has never been reported, but geckos are described as associated with *Belminus peruvianus* (Herrer, Lent and Wygodzinsky, 1954), *Parabelminus yurupucu* (Lent & Wygodzinsky, 1979) and *Triatoma guasayana* (Wygodzinsky & Abalos, 1949) [74]; also, *Hemidactylus frenatus* (Duméril & Bibron, 1836) is a host of *R. prolixus* [75]. In the current study, no nests were found in the explored palms, but one bat was observed in a *Copernicia alba*, and four *R. stali* nymphs were captured in this palm. So, bats could also be a host for this species.

Triatominae were captured regardless of the environment type. Based on occupancy models, palms surrounded by pastures were 2.5-fold more likely to be infested than palms in the forest, and palms at the border between human settlements and forest were 2.9-fold more likely to be infested than palms inside the forest. A total of 48 human blood meals were detected in Triatominae in the three localities where the analysis was conducted: San Borja (SB2 and SB3), San Joaquin (SJ3) and Trinidad (T1). These blood meals were found in 47 nymphal instars, and one female, in both *Rhodnius* species. Looking in detail at the land use of these capture points, SB3 can be defined as a peridomicile—sylvatic (forest) area inside a Tsimané village (indigenous people of the lowlands of Bolivia), and SJ3 as peridomicile in a small brickyard neighborhood surrounded by forest. At these two capture points, the number of captured insects was high, and the smallest palm-to-building distance was 7 and 9 m, respectively. On the contrary, SB2 and T1 were in a wilder environment: a forest fragment (SB2) and a small forest fragment among pastures (T1). In both cases, a ranch was not very far away, at 41 and 58 m, respectively. This is not the first time that human blood meals have been discovered in wild nymphs of *Rhodnius*: for example, 7% of the blood meal detected in *R. pallescens* captured in palms in Panama had a human origin [76]; in Ecuador, 41.5–57.3% of *R. ecuadoriensis* found in the sylvatic environment had fed on human blood, half of them being nymphs [77]. These authors also showed multiple sources of blood meal in younger nymphal instars and hypothesized that cleptohematophagy (bug feeding on the blood meal ingested by another bug) had a role to play. This behavior may explain the human blood meals in nymphal instars while they can only move by walking. Adults, on the other hand, can fly several hundred meters and sometimes kilometers [78]; hence a distance of < 60 m is not that far to go and return to the forest fragment. Another explanation is that humans rest at the base of the palms so that the nymphs can reach them and return to the palm crown; although plausible, this explanation is probably less likely.

## Conclusions

To what extent does *Rhodnius* represent a danger to the human population in the Amazon region of Bolivia? First, it is well-known that palms near dwellings represent a risk because they can be inhabited by *Rhodnius* populations that can invade human dwellings [16]. One of the most common palms in peridomicile area in Bolivia is Motacú (*A. princeps*, *A. phalerata*), a frequently infested palm. In this study, the odds of infestation of *A. phalerata* are particularly high (OR = 71.6 compared to *A. speciosa* and 27.8 compared to *A. princeps*); the OR was only 1.55 compared to *A. speciosa* in [16]. It should be kept in mind that in our study the number of palms was limited. Furthermore, the non-independence of samples within a capture site can lead to an overestimation of the prevalence of infestation and to an underestimation of covariate effects [16]. In the present study, *Rhodnius* was found in every locality, showing a wide distribution. With a *T. cruzi* infection rate of around 30%, these *Rhodnius* populations definitively represent a risk of being a *T. cruzi* vector*,* as their contact with the human population was clearly indicated by the identification of human blood meals. In addition, *R. stali* has been described as a vector in the Alto Beni region, being in a process of domiciliation in peridomicile [10], and *R. robustus* was recently found invading dwellings in the tropics of Cochabamba [22]. At the time this study was conducted, a serological diagnosis of Chagas disease was organized in these six localities [32]. It was noted at that time that the local populations had little knowledge about Chagas disease. The results of that survey also showed that the seropositivity of Chagas disease was 4.1% in Cobija (49 analyzed persons), 6.7% in Riberalta (60 persons analyzed), 6.0% in Guayaramerín (83 persons tested), 5.5% in Trinidad (55 persons analyzed), 2.1% in San Joaquin (48 persons tested) and 11.6% in San Borja (43 people tested). These results clearly show that cases of Chagas disease exist in the Bolivian Amazon. They may be due to local vector or oral transmissions, the latter being frequent in this region [19, 20, 79]. They may also be imported cases, as the Amazon region is a region of intense migration of people who previously lived in areas endemic for Chagas disease [2]. There is an urgent need to develop research to improve the assessment of the local transmission risks and to understand both the modes of transmission and the biological and anthropological factors related to these transmissions in order to generate surveillance and control policies if necessary.

## Supplementary Information

**Additional file 1: Text S1.** Methodology used for the different molecular characterization of Triatominae, blood source meal and *Trypanosoma*. **Table S1.** Description of primers used in molecular characterization of triatomines, blood source meals and *Trypanosoma*. **Table S3.** Distribution of the insects by capture points and developmental stages. **Figure S1.** **a** Environmental description of capture points in Trinidad, San Joaquin and San Borja. **b** Environmental description of capture points in Riberalta, Guayaramerín and Cobija. **Table S4.** General description of the environment around each capture point inside a circle of 500 m in radius. **Table S5.** Description of the haplotypes found for the *Cytb*, *16S*, and *28S-D2* gene fragments. **Figure S2.** Maximum likelihood tree constructed with the 17 *Cytb* haplotypes jointly with < 100% coverage sequences deposited in GenBank. **Figure S3.** Maximum likelihood tree constructed with the eight *16S* haplotypes jointly with sequences deposited in GenBank. **Figure S4.** Maximum likelihood tree constructed with the four *28S-D2* haplotypes jointly with sequences deposited in GenBank. **Figure S5.** Agarose gel electrophoresis (3% agarose) of PCR-amplified products using mini-exon multiplex PCR (MMPCR).

**Additional file 2: Table S2.** Details on analyses and results obtained for each captured insect: capture location, species determination, blood meal origin, and infection by *Trypanosoma*.

## Data Availability

All the sequences associated with Triatominae (*Cytb*,* 16S*,* D2-28S*) described in the present study were deposited in GenBank. The accession numbers are given in Additional file 2: Table S2. All available data on the descriptions of Triatominae specimens and their capture location are given in Additional file 2: Table S2.

## Abbreviations

*Cytb*: Cytochrome B gene
DTU: Discrete typing unit
N1–N5: Nymphal stages 1–5
PCR: Polymerase chain reaction
*16S*: *large* ribosomal subunit 16S RNA gene (LSU-rRNA)
*28S-D2*: *D2* variable region of the *28S* RNA gene

## References

[1] WHO. Chagas disease (American trypanosomiasis). 2020. https://www.who.int/news-room/fact-sheets/detail/chagas-disease-(american-trypanosomiasis). Accessed 3 Jan 2022.

[2] Coura JR, Junqueira AC (2012). Risks of endemicity, morbidity and perspectives regarding the control of Chagas disease in the Amazon region. Mem Inst Oswaldo Cruz.

[3] Coura JR, Viñas PA, Junqueira AC (2014). Ecoepidemiology, short history and control of Chagas disease in the endemic countries and the new challenge for non-endemic countries. Mem Inst Oswaldo Cruz.

[4] Dias JCP, Prata A, Schofield CJ (2002). Chagas disease in the Amazon: an overview of the current situation and perspectives for prevention. Rev Soc Bras Med Trop.

[5] Aguilar HM, Abad-Franch F, Dias JCP, Junqueira ACV, Coura JR (2007). Chagas disease in the Amazon region. Mem Inst Oswaldo Cruz.

[6] Ibisch P, Mérida G, editors. Biodiversidad—riqueza de Bolivia. Santa Cruz de la Sierra: FAN; 2003. https://www.researchgate.net/publication/328028130_Biodiversidad_-_riqueza_de_Bolivia. Accessed 28 June 2021.

[7] Navarro G, Maldonado M (2006). Geografia ecologica de Bolivia.

[8] De la Riva J, Matias A, Torrez M, Martínez E, Dujardin JPP, Martinez E (2001). Adult and nymphs of *Microtriatoma trinidadensis* (Lent, 1951) (Hemiptera: Reduviidae) caught from peridomestic environment in Bolivia. Mem Inst Oswaldo Cruz.

[9] Depickère S, Durán P, López R, Martínez E, Chávez T (2012). After five years of chemical control: colonies of the triatomine *Eratyrus mucronatus* are still present in Bolivia. Acta Trop.

[10] Depickère S, Martinez E, López R, Durán P, Lardeux F, Chavez T (2007). *Rhodnius stali*: a new vector of Chagas disease in Bolivia.

[11] Justi SA, Noireau F, Cortez MR, Monteiro FA (2010). Infestation of peridomestic Attalea phalerata palms by *Rhodnius stali*, a vector of *Trypanosoma cruzi* in the Alto Beni, Bolivia. Trop Med Int Health.

[12] Martínez E, Durán P, López R, Chávez T, Aliaga C, Lardeux F (2012). *Trypanosoma cruzi* I transmitido por *Rhodnius stali* en un foco emergente del trópico de La Paz. Molecular epidemiology and evolution of infectious diseases in Latin America.

[13] Matias A, De la Riva J, Martinez E, Torrez M, Dujardin JP (2003). Domiciliation process of *Rhodnius stali* (Hemiptera: Reduviidae) in Alto Beni, La Paz, Bolivia. Trop Med Int Health.

[14] Matias A, De la Riva JX, Torrez M, Dujardin JP (2001). *Rhodnius robustus* in Bolivia identified by its wings. Mem Inst Oswaldo Cruz.

[15] Rojas Cortez ME. Triatominos de Bolivia y la enfermedad de Chagas. La Paz: Ministerio de Salud y Deportes, Unidad de Epidemiologia, Programa Nacional de Chagas; 2007.

[16] Abad-Franch F, Lima MM, Sarquis O, Gurgel-Gonçalves R, Sánchez-Martín M, Calzada J (2015). On palms, bugs, and Chagas disease in the Americas. Acta Trop.

[17] Abad-Franch F, Palomeque FS, Aguilar HMV, Miles MA (2005). Field ecology of sylvatic *Rhodnius* populations (Heteroptera, Triatominae): risk factors for palm tree infestation in western Ecuador. Trop Med Int Health.

[18] Miles MA, Arias JR, De Souza AA (1983). Chagas’ disease in the Amazon basin: V. Periurban palms as habitats of *Rhodnius robustus* and *Rhodnius pictipes*—triatomine vectors of Chagas’ disease. Mem Inst Oswaldo Cruz.

[19] Vilca Ugaz JA. Descripcion del brote de la enfermedad de Chagas agudo por posible transmision oral ocurido en el Municipio de Guayanamerin en 2010. 2011. pp 75. http://atlas.umss.edu.bo:8080/xmlui/handle/123456789/125

[20] Santalla Vargas J, Oporto Carrasco P, Espinoza E, Rios T, Brutus L (2011). First reported outbreak of Chagas disease in the Bolivian Amazonean zone: a report of 14 cases of oral transmission of acute *Trypanosoma cruzi* in Guayaramerín, Beni-Bolivia. Biofarbo.

[21] Brenière SF, Condori EW, Buitrago R, Sosa LF, Macedo CL, Barnabé C (2017). Molecular identification of wild triatomines of the genus *Rhodnius* in the Bolivian Amazon: strategy and current difficulties. Infect Genet Evol.

[22] Rojas-Cortez M, Pinazo M-J, Garcia L, Arteaga M, Uriona L, Gamboa S (2016). *Trypanosoma cruzi*-infected *Panstrongylus geniculatus* and *Rhodnius robustus* adults invade households in the tropics of Cochabamba region of Bolivia. Parasit Vectors.

[23] Monteiro FA, Weirauch C, Felix M, Lazoski C, Abad-Franch F (2018). Evolution, systematics, and biogeography of the triatominae, vectors of Chagas disease. Adv Parasitol.

[24] Zhao Y, Galvão C, Cai W (2021). *Rhodnius micki*, a new species of Triatominae (Hemiptera, Reduviidae) from Bolivia. ZooKeys.

[25] Justi SA, Galvão C (2017). The evolutionary origin of diversity in Chagas disease vectors. Trends Parasitol.

[26] Korshunova T, Picton B, Furfaro G, Mariottini P, Pontes M, Prkić J (2019). Multilevel fine-scale diversity challenges the ‘cryptic species’ concept. Sci Rep.

[27] Brito RN, Geraldo JA, Monteiro FA, Lazoski C, Souza RCM, Abad-Franch F (2019). Transcriptome-based molecular systematics: *Rhodnius montenegrensis* (Triatominae) and its position within the *Rhodnius prolixus*–*Rhodnius robustus* cryptic-species complex. Parasit Vectors.

[28] Lázari Cacini G, de Oliveira J, Belintani T, dos Santos SÉ, Olaia N, Pinto MC (2022). Immature instars of three species of Rhodnius Stål, 1859 (Hemiptera, Reduviidae, Triatominae): morphology, morphometry, and taxonomic implications. Parasit Vectors.

[29] Abad-Franch F, Monteiro FA (2007). Biogeography and evolution of Amazonian triatomines (Heteroptera: Reduviidae): implications for Chagas disease surveillance in humid forest ecoregions. Mem Inst Oswaldo Cruz.

[30] Monteiro FA, Barrett TV, Fitzpatrick S, Cordon-Rosales C, Feliciangeli D, Beard CB (2003). Molecular phylogeography of the Amazonian Chagas disease vectors *Rhodnius prolixus* and *R. robustus*. Mol Ecol.

[31] Nascimento J, Da Rosa J, Salgado-Roa F, Hernández C, Pardo-Diaz C, Alevi KCC (2019). Taxonomical over splitting in the *Rhodnius prolixus* (Insecta: Hemiptera: Reduviidae) clade: are *R. taquarussuensis* (da Rosa et al., 2017) and *R. neglectus* (Lent, 1954) the same species?. PLoS ONE.

[32] Callapa J, Linares M, Paucara M, Cerruto Y, Romero N, Suxo Y (2018). Epidemiological, serological and molecular study of the Chagas disease in the Bolivian Amazon. Rev Con-Cienc.

[33] Revelle W. psych: procedures for personality and psychological research. Evanston: Northwestern University; 2017. https://cran.r-project.org/package=psych. Accessed 2 Apr 2021.

[34] Lê S, Josse J, Husson F (2008). FactoMineR: a package for multivariate analysis. J Stat Softw.

[35] Slowikowski K. ggrepel: automatically position non-overlapping text labels with “ggplot2.” 2020. https://cran.r-project.org/package=ggrepel. Accessed 18 Mar 2021.

[36] R Core Team (2016). R: a language and environment for statistical computing.

[37] Kassambara A. Practical guide to principal component methods in R: PCA, M (CA), FAMD, MFA, HCPC, factoextra. STHDA; 2017. https://www.datanovia.com/en/product/practical-guide-to-principal-component-methods-in-r/. Accessed 5 Mar 2021.

[38] MacKenzie DI, Nichols JD, Lachman GB, Droege S, Andrew Royle J, Langtimm CA (2002). Estimating site occupancy rates when detection probabilities are less than one. Ecology.

[39] MacKenzie DI, Nichols J, Royle J, Pollock K, Bailey L, Hines J. Occupancy estimation and modeling—inferring patterns and dynamics of species occurrence, 2nd edition. London: Academic Press; 2017. https://www.elsevier.com/books/occupancy-estimation-and-modeling/mackenzie/978-0-12-407197-1.

[40] Hines JE. PRESENCE—software to estimate patch occupancy and related parameters. USGS-PWRC; 2006. http://www.mbr-pwrc-usgs.gov/software/presence.html. Accessed 15 Oct 2021.

[41] Noireau F, Flores R, Vargas F (1999). Trapping sylvatic Triatominae (Reduviidae) in hollow trees. Trans R Soc Trop Med Hyg.

[42] Lent H, Jurberg J, Galvão C (1993). *Rhodnius stali* N. SP., afim de *Rhodnius pictipes* Stal, 1872 (Hemiptera, Reduviidae, Triatominae). Mem Inst Oswaldo Cruz.

[43] Lent H, Wygodzinsky P (1979). Revision of the Triatominae (Hemiptera, Reduviidae), and their significance as vectors of Chagas’ disease. Bull Am Mus Nat Hist.

[44] Aljanabi SM, Martinez I (1997). Universal and rapid salt-extraction of high quality genomic DNA for PCR-based techniques. Nucleic Acids Res.

[45] Weirauch C, Munro JB (2009). Molecular phylogeny of the assassin bugs (Hemiptera: Reduviidae), based on mitochondrial and nuclear ribosomal genes. Mol Phylogenet Evol.

[46] Fitzpatrick S, Feliciangeli MD, Sanchez-Martin MJ, Monteiro FA, Miles MA. Molecular genetics reveal that silvatic *Rhodnius prolixus* do colonise rural houses. PLoS Negl Trop Dis. 2008;2(4):e210. 10.1371/journal.pntd.0000210. 10.1371/journal.pntd.0000210 PMC2270345 18382605

[47] Buitrago R, Bosseno M-F, Depickère S, Waleckx E, Salas R, Aliaga C (2016). Blood meal sources of wild and domestic *Triatoma infestans* (Hemiptera: Reduviidae) in Bolivia: connectivity between cycles of transmission of *Trypanosoma cruzi*. Parasit Vectors.

[48] Lauthier JJ, Tomasini N, Barnabé C, Monje Rumi MM, Alberti D’Amato AM, Ragone PG (2012). Candidate targets for multilocus sequence typing of *Trypanosoma cruzi*: validation using parasite stocks from the Chaco region and a set of reference strains. Infect Genet Evol.

[49] Messenger LA, Llewellyn MS, Bhattacharyya T, Franzén O, Lewis MD, Ramírez JD (2012). Multiple mitochondrial introgression events and heteroplasmy in *Trypanosoma cruzi* revealed by maxicircle MLST and next generation sequencing. PLoS Negl Trop Dis.

[50] Aliaga C, Brenière SF, Barnabé C (2011). Further interest of miniexon multiplex PCR for a rapid typing of *Trypanosoma cruzi* DTU groups. Infect Genet Evol.

[51] Tamura K, Stecher G, Peterson D, Filipski A, Kumar S (2013). MEGA6: molecular evolutionary genetics analysis version 6.0. Mol Biol Evol.

[52] Librado P, Rozas J (2009). DnaSP v5: a software for comprehensive analysis of DNA polymorphism data. Bioinformatics.

[53] Nguyen L-T, Schmidt HA, von Haeseler A, Minh BQ (2015). IQ-TREE: a fast and effective stochastic algorithm for estimating maximum-likelihood phylogenies. Mol Biol Evol.

[54] Kalyaanamoorthy S, Minh BQ, Wong TKF, von Haeseler A, Jermiin LS (2017). ModelFinder: fast model selection for accurate phylogenetic estimates. Nat Methods.

[55] Hoang DT, Chernomor O, von Haeseler A, Minh BQ, Vinh LS (2018). UFBoot2: improving the ultrafast bootstrap approximation. Mol Biol Evol.

[56] Yu G, Smith DK, Zhu H, Guan Y, Lam TT-Y (2017). ggtree: an R package for visualization and annotation of phylogenetic trees with their covariates and other associated data. Methods Ecol Evol.

[57] Wang L-G, Lam TT-Y, Xu S, Dai Z, Zhou L, Feng T (2020). Treeio: an R package for phylogenetic tree input and output with richly annotated and associated data. Mol Biol Evol.

[58] Bandelt HJ, Forster P, Röhl A (1999). Median-joining networks for inferring intraspecific phylogenies. Mol Biol Evol.

[59] Leigh JW, Bryant D (2015). popart: full-feature software for haplotype network construction. Methods Ecol Evol.

[60] D’Alessandro A, Eberhard M, de Hincapie O, Halstead S (1986). *Trypanosoma Cruzi* and *Trypanosoma Rangeli* in *Saimiri Sciureus* from Bolivia and *Saguinus Mistax* from Brazil. Am J Trop Med Hyg.

[61] Grijalva MJ, Suarez-Davalos V, Villacis AG, Ocaña-Mayorga S, Dangles O (2012). Ecological factors related to the widespread distribution of sylvatic *Rhodnius ecuadoriensis* populations in southern Ecuador. Parasit Vectors.

[62] Pineda V, Montalvo E, Alvarez D, Santamaría AM, Calzada JE, Saldaña A (2008). Feeding sources and trypanosome infection index of *Rhodnius pallescens* in a Chagas disease endemic area of Amador County, Panama. Rev Inst Med Trop São Paulo.

[63] Meneguetti DUDO, Soares EB, Campaner M, Camargo LMA, Meneguetti DUDO, Soares EB (2014). First report of *Rhodnius montenegrensis* (Hemiptera: Reduviidae: Triatominae) infection by *Trypanosoma rangeli*. Rev Soc Bras Med Trop.

[64] Castro GVDS, Ribeiro MAL, Ramos LJ, de Oliveira J, da Rosa JA, Camargo LMA (2017). *Rhodnius stali*: new vector infected by *Trypanosoma rangeli* (Kinetoplastida, Trypanosomatidae). Rev Soc Bras Med Trop.

[65] Ballesteros-Rodea G, Martinez Cuevas TI, Jiménez Ramos B, Antonio CA (2018). Chagas disease: an overview of diagnosis. J Microbiol Exp.

[66] Muscarella R, Emilio T, Phillips OL, Lewis SL, Slik F, Baker WJ (2020). The global abundance of tree palms. Glob Ecol Biogeogr.

[67] ter Steege H, Pitman NCA, Sabatier D, Baraloto C, Salomão RP, Guevara JE, et al. Hyperdominance in the Amazonian tree flora. Science. 2013;342:1243092. 10.1126/science.1243092. 10.1126/science.1243092 24136971

[68] Cámara-Leret R, Faurby S, Macía MJ, Balslev H, Göldel B, Svenning J-C (2017). Fundamental species traits explain provisioning services of tropical American palms. Nat Plants.

[69] Calderón JM, González C (2020). Co-occurrence or dependence? Using spatial analyses to explore the interaction between palms and *Rhodnius* triatomines. Parasit Vectors.

[70] Carcavallo RV, Girón IG, Jurberg J, Lent H. Atlas of Chagas’ disease vectors in the Americas. Editora Fiocruz: Rio de Janeiro; 1998.

[71] da Rosa JA, Rocha CS, Sueli G, Cristina PM, Mendonça VJ, Filho JCRF (2012). Description of *Rhodnius montenegrensis* n. sp. (Hemiptera: Reduviidae: Triatominae) from the state of Rondônia, Brazil. Zootaxa.

[72] Urbano P, Poveda C, Molina J (2015). Effect of the physiognomy of *Attalea butyracea* (Arecoideae) on population density and age distribution of *Rhodnius prolixus* (Triatominae). Parasit Vectors.

[73] Jansen AM, Xavier SCDC, Roque ALR (2018). *Trypanosoma cruzi* transmission in the wild and its most important reservoir hosts in Brazil. Parasit Vectors.

[74] Georgieva AY, Gordon ERL, Weirauch C (2017). Sylvatic host associations of Triatominae and implications for Chagas disease reservoirs: a review and new host records based on archival specimens. PeerJ.

[75] Calderón JM, Erazo D, Kieran TJ, Gottdenker NL, León C, Cordovez J (2020). How microclimatic variables and blood meal sources influence *Rhodnius prolixus* abundance and *Trypanosoma cruzi* infection in *Attalea butyracea* and *Elaeis guineensis* palms?. Acta Trop.

[76] Kieran TJ, Gottdenker NL, Varian CP, Saldaña A, Means N, Owens D (2017). Blood meal source characterization using illumina sequencing in the Chagas disease vector *Rhodnius pallescens* (Hemiptera: Reduviidae) in Panamá. J Med Entomol.

[77] Ocaña-Mayorga S, Bustillos JJ, Villacís AG, Pinto CM, Brenière SF, Grijalva MJ (2021). Triatomine feeding profiles and *Trypanosoma cruzi* infection, implications in domestic and sylvatic transmission cycles in Ecuador. Pathogens.

[78] Castro LA, Peterson JK, Saldaña A, Perea MY, Calzada JE, Pineda V (2014). Flight behavior and performance of *Rhodnius pallescens* (Hemiptera: Reduviidae) on a tethered flight mill. J Med Entomol.

[79] dos Santos VRC, de Meis J, Savino W, Andrade JAA, Vieira JRDS, Coura JR, et al. Acute Chagas disease in the state of Pará, Amazon region: is it increasing? Mem Inst Oswaldo Cruz. 2018;113:e170298. 10.1590/0074-02760170298. 10.1590/0074-02760170298 PMC5951676 29742200

