# Supplementary material for: Rhodnius (Stål, 1859) (Hemiptera, Triatominae) genus in Bolivian Amazonia: a risk for human populations?
Source: Parasit Vectors. 2022 Aug 29;15:307. doi: 10.1186/s13071-022-05423-3 (PMC9426019; doi:10.1186/s13071-022-05423-3)

Additional file 1

Item	Page
**Text S1.** Methodology used for the different molecular characterization of Triatominae, blood source meal and *Trypanosoma*.	**2**
**Table S1**. Description of primers used in molecular characterization of triatomines, blood source meals, and *Trypanosoma*.	**3**
**Table S3**. Distribution of the insects by capture points and developmental stages.	**4**
**Figure S1.a**: Environmental description of capture points in Trinidad, San Joaquin and San Borja.	**5**
**Figure S1.b**: Environmental description of capture points in Riberalta, Guayaramerín and Cobija.	**6**
**Table S4**. General description of the environment around each capture point inside a circle of 500 m in radius.	**7**
**Table S5**. Description of the haplotypes found for the *Cytb*, *16S*, and *28S-D2* gene fragments.	**8-9**
**Figure S2**. Maximum likelihood tree constructed with the 17 *Cytb* haplotypes jointly with < 100% coverage sequences deposited in GenBank.	**10**
**Figure S3**. Maximum likelihood tree constructed with the eight *16S* haplotypes jointly with sequences deposited in GenBank.	**11**
**Figure S4**. Maximum likelihood tree constructed with the four *28S-D2* haplotypes jointly with sequences deposited in GenBank.	**12**
**Figure S5**. Agarose gel electrophoresis (3% agarose) of PCR amplified products using mini-exon multiplex PCR (MMPCR).	**13**

Table S2 corresponds to Additional file 2: Table S2

Text S1. Methods

a) DNA extraction (rapid salt-extraction method)
Briefly, after grinding each biological material, 200 µl of Tris 10 mM-NaCl 0.4M-EDTA 2mM buffer, 20 µl of SDS 20%, and 10 µl of proteinase K at 20 mg/ml were added, and the mixture was incubated overnight at 37° C. Then, the DNA extraction was performed in NaCl 6M, followed by isopropanol DNA precipitation and finally 75% ethanol washing. DNA samples were dried and diluted in 30 µl (N1-N2), 50 µl (N3-N4), or 80 µl (N5-adults) of AE buffer (Qiagen, Courtaboeuf, France) for triatomine DNAs, or in 30 µl for the digestive tract. DNA concentration was determined by measuring the optical density at 260 nm using a NanoDrop 1000 Spectrophotometer V3.7 (Thermo Fisher Scientific, Wilmington, DE, USA).

b) Molecular characterization of triatomines
DNA was amplified in a 50 µl reaction volume containing 25 µl of 2 X GoTaq Green Master Mix solution (Promega, Madison, USA), 1.25 pmol of each primer, and 5 ng of DNA template in a thermocycler (Bio-Rad, Hercules, CA, USA) under the following conditions: one step at 95°C for 5 min followed by 35 cycles: 95°C for 30 s, 47°C for 30 s, 72°C for 1 min, and a final elongation step at 72°C for 5 min. Ten microliters of each PCR product were analyzed by electrophoresis in a 1% agarose gel using SYBR Safe (Fisher Scientific, Illkirch, France) to stain DNA and visualized under UV light.

c) Molecular characterization of blood source meals
The amplification was realized in a 50 µl reaction volume containing 25 µl 2 X GoTaq Green Master Mix (Promega, Madison, USA), 2 pmol of each primer and 10 µg of DNA template in a thermocycler (Bio-Rad, Hercules, CA, USA) under the following conditions: one step at 95°C for 3.5 min followed by 36 cycles: 95°C for 30 s, 55°C for 50 s, 72°C for 40 s, and a final elongation step at 72°C for 5 min [44]. Ten microliters of each PCR product were analyzed by electrophoresis in a 1% agarose gel using SYBR Safe (Fisher Scientific, Illkirch, France) to stain DNA and visualized under UV light.

d) Molecular characterization of *Trypanosoma* and DTUs detection
The amplification was performed in a 50 µl reaction volume containing 1 µg of DNA; 1 µM of each primer, 1 U of Taq DNA polymerase, 5 µl of 10x buffer, and a 0.5 µl [5 mM] dNTPs in a thermocycler under the following conditions: one step at 94°C for 1 min followed by 35 cycles: 94°C for 30 s, 55°C for 30 s, 72°C for 30 s, and a final elongation step at 72°C for 5 min.

e) Methodology for the mini-exon multiplex PCR (MMPCR) for the characterization of *Trypanosoma* and DTUs detection
DNA amplification was performed in 25 µl reaction volume containing 12.5 µl of 2 X GoTaq Green Master Mix (Promega, Charbonnières-les-Bains, France), 0.2 µM of each primer, and 5 µl of DNA template in a thermocycler (Bio-Rad, Hercules, CA, USA) under the following conditions: one step at 94°C for 5 min followed by 35 cycles: 94°C for 30 s, 50°C for 30 s, 72°C for 30 s, and a final elongation step at 72°C for 7 min. Ten microliters of each PCR product were analyzed by electrophoresis in a 3% agarose gel using SYBR SafeDNA gel stain and visualized under UV light.

Table S1. Description of primers used in molecular characterization of triatomines, blood source meals, and *T. cruzi* DTUs.

Gene	Forward	Reverse	Reference
	Triatomine species		
16S (LSU-rRNA)	16sa (5'-CGC CTG TTT ATC AAA AAC AT-3')	16sb (5'-CTC CGG TTT GAA CTC AGA TCA-3')	1
Cytb	Cytb7432F (5'-GGA CGW GGW ATT TAT TAT GGA TC-3')	Cytb7433R (5'-GCW CCA ATT CAR GTT ART AA-3')	2
28S-D2	D2F (5'-GCG AGT CGT GTT GCT TGA TAG TGC AG-3')	D2R (5'-TTG GTC CGT GTT TCA AGA CGG G-3')	3
	Blood meal source		
Cytb	5'-CCC CTC AGA ATG ATA TTT GTC CTC A-3'	5'-CCA TCC AAC ATC TCA GCA TGA TGA AA-3'	4
	Trypanosoma cruzi		
GPI	Gpi-L: 5'-CGC CAT GTT GTG AAT ATT GG-3'	Gpi-R: 5'-GGC GGA CCA CAA TGA GTA TC-3'	5
GPX	Gp-L: 5'-CGT GGC ACT CTC CAA TTA CA-3'	Gp-R: 5'-AAT TTA ACC AGC GGG ATG C-3'	5
HMCOAR	CoAr-L: 5'-AGG AGG CTT TTG AGT CCA CA-3'	CoAr-R: 5'-TCC AAC AAC ACC AAC CTC AA-3'	5
LAP	Lap-1: 5'-TGT ACA TGT TGC TTG GCT GAG-3'	Lap-2: 5'-GCT GAG GTG ATT AGC GAC AAA-3'	5
PDH	Pdh-L: 5'-GGG GCA AGT GTT TGA AGC TA-3'	Pdh-R: 5'-AGA GCT CGC TTC GAG GTG TA-3'	5
COII	CoII Fwd: 5'-GTT ATT ATC TTT TGT TTG TTT TGT GTG-3'	COII Rvs: 5'-AAC AAT TGG CAT AAA TCC ATG T-3'	6

1. Weirauch et al. Mol Phylogenet Evol. 2009;53: 287–299. doi:10.1016/j.ympev.2009.05.039
2. Monteiro et al. Mol Ecol. 2003;12: 997–1006.
3. Fitzpatrick et al. PLoS Negl Trop Dis. 2008;2. doi:10.1371/journal.pntd.0000210
4. Buitrago et al. Parasit Vectors. 2016;9: 214. doi:10.1186/s13071-016-1499-0
5. Lauthier et al. Infect Genet Evol. 2012;12: 350–358. doi:10.1016/j.meegid.2011.12.008
6. Aliaga et al. Infect Genet Evol. 2011;11: 1155–1158. doi:10.1016/j.meegid.2010.11.013

Table S3. Distribution of the insects by capture points and developmental stages.

Locality	N1	N2	N3	N4	N5	Male	Female	Total
San Borja 2 (SB2)	4	-	-	-	-	1*	2*	7
San Borja 3 (SB3)	18	11	24	9	8	2**	3**	75
Total San Borja	22	11	24	9	8	3	5	82
San Joaquin 1 (SJ1)	-	-	-	2	-	-	-	2
San Joaquin 3 (SJ3)	21	18	26	19	14	-	-	98
Total San Joaquin	21	18	26	21	14	-	-	100
Trinidad 1 (T1)	34	26	30	9	6	-	-	105
Trinidad 2 (T2)	1	-	2	1	2	-	-	6
Total Trinidad	35	26	32	10	8	-	-	111
Cobija 1 (C1)	-	4	1	3	3	1*	-	12
Cobija 2 (C2)	-	-	-	-	2	-	-	2
Cobija 3 (C3)	2	-	-	-	1	-	-	3
Total Cobija	2	4	1	3	6	1	-	17
Guayaramerín 1 (G1)	1	-	-	-	2	-	-	3
Guayaramerín 2 (G2)	-	-	-	-	3	-	-	3
Total Guayaramerín	1	-	-	-	5	-	-	6
Riberalta 1 (R1)	-	1	1	-	-	-	-	2
Riberalta 2 (R2)	1	-	3	2	1	-	-	7
Total Riberalta	1	1	4	2	1			9
TOTAL	82	60	86	45	42	4	5	325

*: identified as *R. robustus* by morphological keys;
**: identified as *R. robustus* or *R. stali* by morphological keys

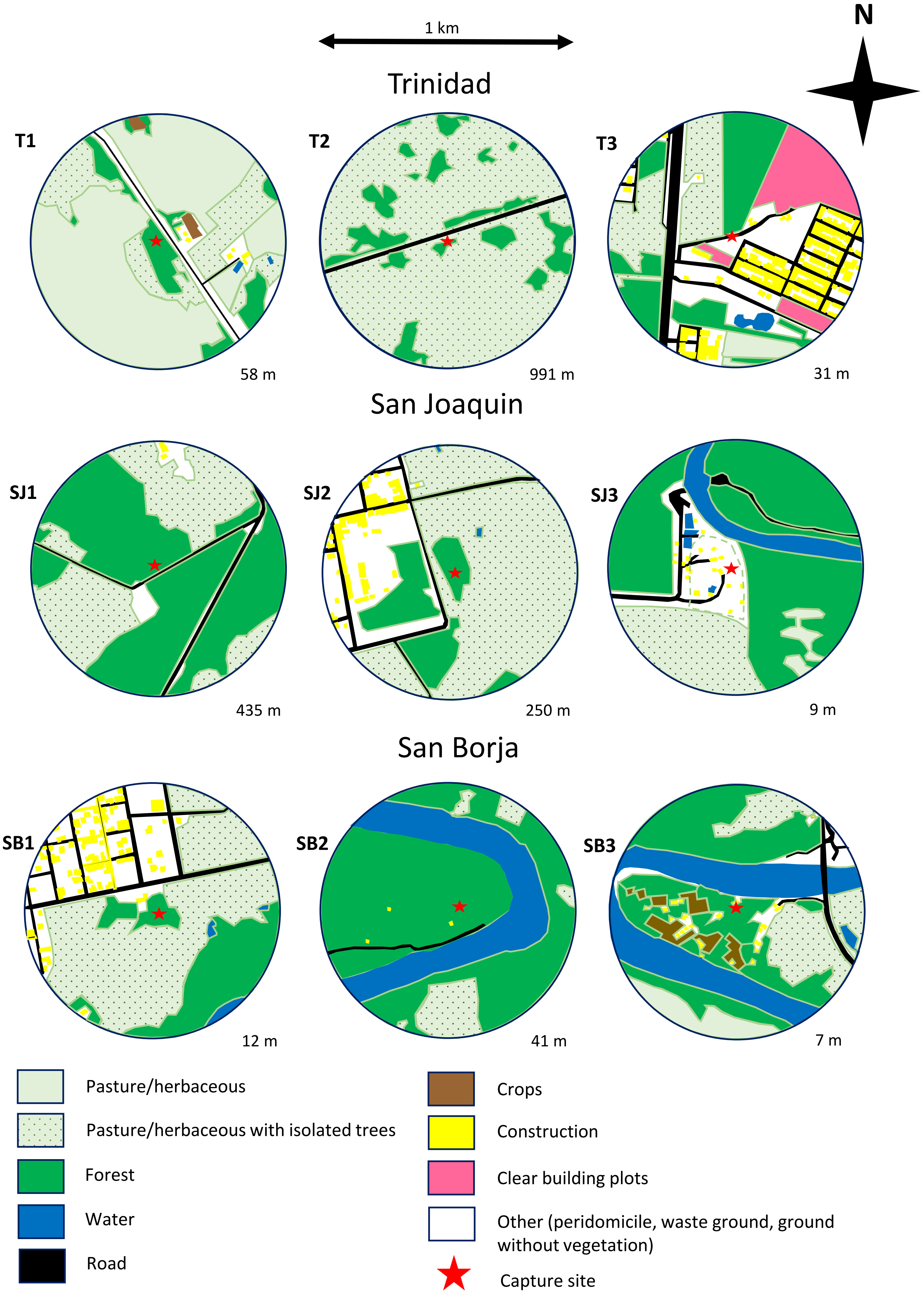

Figure S1.a: Environmental description of capture points in Trinidad, San Joaquin and San Borja. Distance (bottom right of each circle) represents the shortest distance traps - closest construction.

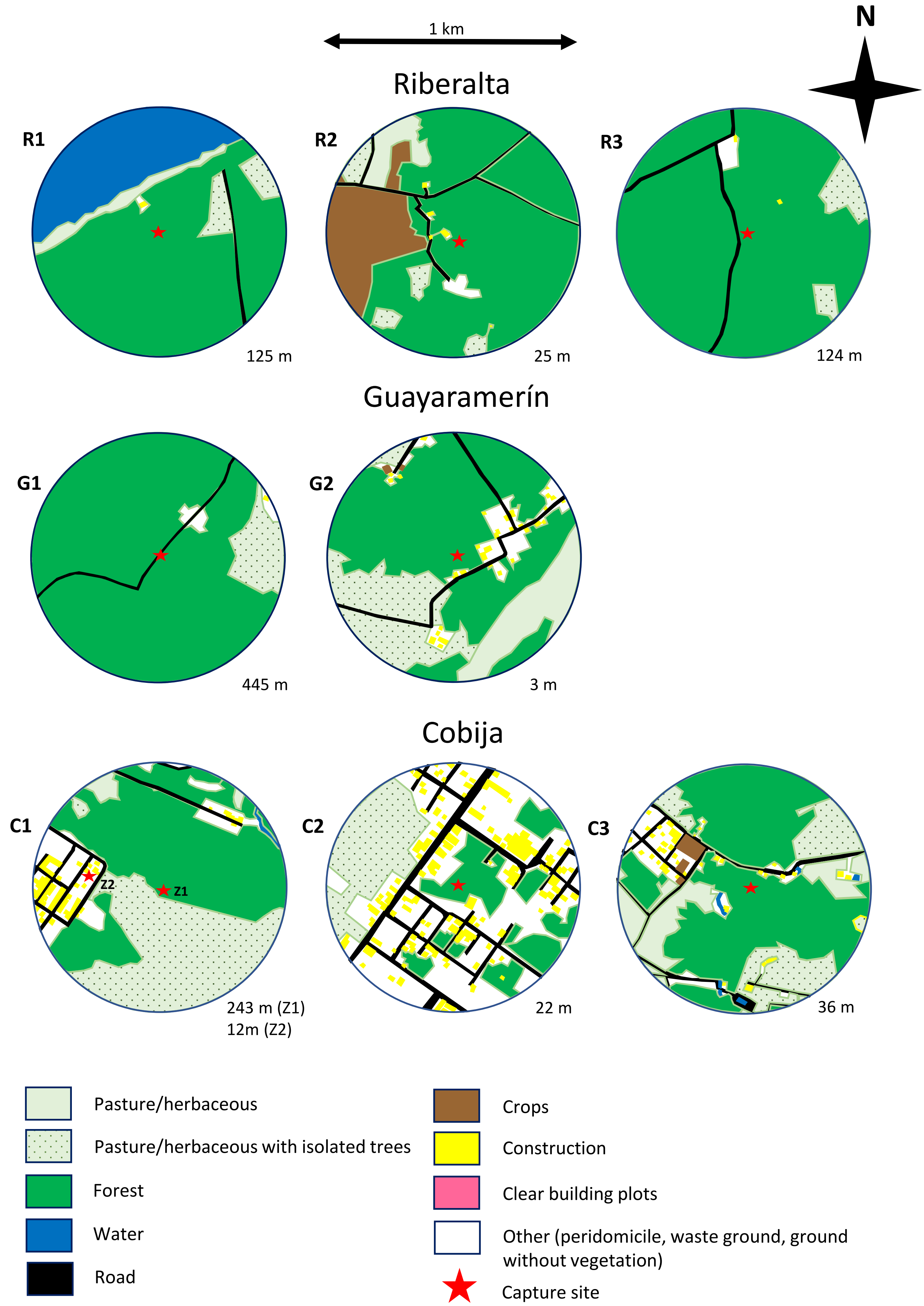

Figure S1.b: Environmental description of capture points in Riberalta, Guayaramerín, and Cobija. Distance (bottom right of each circle) represents the shortest distance traps - closest construction.

Table S4. General description of the environment around each capture point inside a circle of 500 m in radius.

Capture point	General description	Smallest distance*	Land cover description								
			Crop (%)	Forest (%)	Water (%)	Pasture/ herbaceous + trees (%)	Pasture/ herbaceous (%)	Road (%)	Construction (%)	Clear Building plot (%)	Other (%)
SB1	Peridomicile, border of the city	12 m	0.00	19.22	0.97	46.59	0.00	4.21	3.00	0.00	26.01
SB2	Forest fragment near the river, 1.5 km from the city	41 m	0.00	67.09	24.31	7.77	0.00	0.39	0.03	0.00	0.42
SB3	Peridomiciles of an indigenous village, 1 km from the city	7 m	3.97	38.66	24.24	16.14	5.79	1.52	0.31	0.00	9.38
SJ1	Fragment forest, 3.2 km from the village	435 m	0.00	58.47	0.00	33.42	0.00	1.78	0.10	0.00	6.23
SJ2	Fragment forest surrounded by pastures, at the border of the village	250 m	0.00	17.15	0.24	65.15	0.00	3.95	3.13	0.00	10.38
SJ3	Small brickyards neighborhood at 1.6 km from the village	9 m	0.00	54.74	8.40	16.76	2.38	2.74	0.29	0.00	14.68
T1	Fragment forest surrounded by pastures, 13.3 km from the city	58 m	0.72	9.60	0.10	18.80	61.90	1.40	0.12	0.00	7.37
T2	Isolated palm trees surrounded by pastures, 5.5 km from the city	991 m	0.00	14.64	0.00	84.19	0.00	1.17	0.00	0.00	0.00
T3	Newly constructed neighborhood, 2.5 km from the city	31 m	0.00	20.98	0.77	16.69	3.14	9.67	6.66	12.99	29.09
C1	Border of the San Juan neighborhood of Cobija, peridomiciles and forest fragment	12 m	0.00	49.73	0.27	32.80	1.47	2.21	1.63	0.00	11.89
C2	Inside the San Juan neighborhood of Cobija	22 m	0.00	17.48	0.00	16.50	0.00	8.35	6.14	0.00	51.52
C3	Fragment forest, Avaroa community, 4 km from Cobija	36 m	0.91	56.40	0.42	14.67	10.99	10.87	0.93	0.00	4.81
G1	Forest, 29 km from the city	445 m	0.00	91.50	0.00	7.20	0.00	0.52	0.02	0.00	0.76
G2	Forest at the border of Primero de Mayo village, 9.7 km from the city	3 m	0.11	59.33	0.00	19.03	11.76	1.79	0.63	0.00	7.34
R1	Forest near a lake, in Warnes, 10.6 km from the city	125 m	0.00	65.67	25.67	4.23	3.80	0.33	0.05	0.00	0.25
R2	Forest fragment, 2.1 km from the city	25 m	16.96	69.69	0.00	4.21	4.49	0.85	0.11	0.00	3.69
R3	Forest fragment, 13 km from the city	124 m	0.00	88.60	0.00	4.72	0.00	1.38	0.03	0.00	5.26

*Smallest distance corresponds to the shortest distance between traps and the closest construction. The surface represented by each category of land use is expressed in percentage. See Material and Methods for more details.

Table S5. Description of the haplotypes found for the *Cytb*, *16S*, and 28S-*D2* gene fragments.

Gene fragment	Haplotype	Cover	Identity	GenBank accession number	Putative species	No. and place of specimens
Cytb **(612-bp)**	Hap1-Cytb	100%	99.67%	FJ887793.1	*R. robustus*	17<br>• 17 in T1
			99.51%	NC_050328.1	*R. prolixus*	
			99.01%	EF011714.1	*R. robustus*	
	Hap2-Cytb	100%	99.67%	NC_050328.1	*R. prolixus*	1<br>• 1 in T1
			99.67%	FJ887793.1	*R. robustus*	
			99.34%	EF011714.1	*R. robustus*	
			99.18%	EF011709.1	*R. robustus*	
			99.01%	EF011713.1	*R. robustus*	
	Hap3-Cytb	100%	99.84%	FJ887793.1	*R. robustus*	15<br>• 2 in T1<br>• 5 in SB2<br>• 8 in SB3
			99.67%	NC_050328.1	*R. prolixus*	
			99.18%	EF011714.1	*R. robustus*	
	Hap4-Cytb	100%	99.84%	FJ887793.1	*R. robustus*	19<br>• 1 in SJ1<br>• 18 in SJ3
			99.51%	NC_050328.1	*R. prolixus*	
			99.18%	EF011714.1	*R. robustus*	
	Hap5-Cytb	100%	99.34%	FJ887793.1	*R. robustus*	1<br>• 1 in SJ1
			99.01%	NC_050328.1	*R. prolixus*	
	Hap6-Cytb	100%	99.67%	FJ887793.1	*R. robustus*	1<br>• 1 in SJ3
			99.34%	NC_050328.1	*R. prolixus*	
			99.01%	EF011714.1	*R. robustus*	
	Hap7-Cytb	100%	100%	FJ887793.1	*R. robustus*	3<br>• 1 in SB2<br>• 2 in SB3
			99.67%	NC_050328.1	*R. prolixus*	
			99.34%	EF011714.1	*R. robustus*	
			99.01%	EF011713.1	*R. robustus*	
	Hap8-Cytb	100%	99.51%	EF011714.1	*R. robustus*	4<br>• 1 in SB2<br>• 3 in SB3
			99.50%	EF011724.1	*R. robustus*	
			99.34%	EF011711.1,<br>EF011710.1,<br>EF011709.1,<br>AF421341.1	*R. robustus*<br>*R. robustus*<br>*R. robustus*<br>*R. robustus*	
			99.18%	NC_050328.1,<br>FJ887793.1,<br>EF011713.1	*R. prolixus*<br>*R. robustus*<br>*R. robustus*	
	Hap9-Cytb	100%	99.67%	FJ887793.1	*R. robustus*	1<br>• 1 in SB3
			99.51%	NC_050328.1	*R. prolixus*	
			99.01%	EF011714.1<br>EF011724.1	*R. robustus*<br>*R. robustus*	
	Hap10-Cytb	100%	99.67%	EF011714.1	*R. robustus*	1<br>• 1 in SB3
			99.34%	NC_050328.1,<br>FJ887793.1,<br>EF011713.1,<br>EF011720.1	*R. prolixus*<br>*R. robustus*<br>*R. Robustus*<br>*R. robustus*	
			99.18%	EF071583.1,<br>EF011711.1,<br>EF011710.1,<br>EF011709.1,<br>AF421341.1	*R. robustus*<br>*R. robustus*<br>*R. robustus*<br>*R. robustus*<br>*R. robustus*	
	Hap11-Cytb	100%	99.67%	EF011711.1,<br>AF421341.1	*R. robustus*<br>*R. robustus*	11<br>• 5 in C1<br>• 2 in C2<br>• 2 in C3<br>• 2 in G1
			99.50%	EF011724.1	*R. robustus*	
			99.34%	EF011710.1,<br>EF011709.1	*R. robustus*<br>*R. robustus*	
			99.18%	EF011714.1	*R. robustus*	
	Hap12-Cytb	100%	99.83%	EF011724.1	*R. robustus*	4<br>• 1 in C1<br>• 1 in C3<br>• 1 in R1<br>• 1 in R2
			99.67%	EF011711.1,<br>EF011710.1,<br>EF011709.1,<br>AF421341.1	*R. robustus*<br>*R. robustus*<br>*R. robustus*<br>*R. robustus*	
			99.18%	EF011720.1,<br>EF011714.1	*R. robustus*<br>*R. robustus*	
			99.01%	EF071583.1	*R. robustus*	
	Hap13-Cytb	100%	99.84%	EF011714.1	*R. robustus*	2<br>• 2 in C1
			99.51%	NC_050328.1,<br>FJ887793.1,<br>EF011713.1	*R. prolixus*<br>*R. robustus*<br>*R. robustus*	
			99.34%	EF011711.1,<br>EF011710.1,<br>EF011709.1,<br>AF421341.1	*R. robustus*<br>*R. robustus*<br>*R. robustus*<br>*R. robustus*	
			99.18%	EF011720.1	*R. robustus*	
			99.01%	EF071583.1	*R. robustus*	

All sequences with 100% cover and > 99% Identity with GenBank sequences are shown (cover < 100% also shown when Identity is low), giving their Access Number and the correspondent species. The number of individuals with each haplotypes and where they were captured are given. C: Cobija, G: Guayaramerín, R: Riberalta, SB: San Borja, SJ: San Joaquin, T: Trinidad.

Gene fragment	Haplotype	Cover	Identity	GenBank accession number	Putative species	No. and place of specimens
Cytb **(612-bp)**	Hap14-Cytb	100%	99.67%	EF011724.1	*R. robustus*	1<br>• 1 in R2
			99.51%	EF011711.1,<br>EF011710.1,<br>EF011709.1,<br>AF421341.1	*R. robustus*<br>*R. robustus*<br>*R. robustus*<br>*R. robustus*	
			99.01%	EF011720.1,<br>EF011714.1	*R. robustus*<br>*R. Robustus*	
	Hap15-Cytb	100%	100%	FJ887791.1	*R. stali*	4<br>• 4 in T2
			99.84%	FJ887790.1	*R. stali*	
	Hap16-Cytb	100%	96.05%	FJ887790.1	*R. stali*	12<br>• 12 in SB3
		96%	100%	KT805150.1	*R. stali*	
	Hap17-Cytb	100%	98.85%	FJ887791.1	*R. stali*	1<br>• 1 in R1
			98.68%	FJ887790.1	*R. stali*	
16S **(431-bp)**	Hap1-16S	100%	98.83%	NC_050328.1	*R. prolixus*	7<br>• 1 in SB2<br>• 6 in SB3
		69%	100%	KT805173.1	*R. robustus*	
	Hap2-16S	99%	99.70%	NC_050328.1	*R. prolixus*	1<br>• 1 in SJ3
		78%	99.11%	AF045705.2	*R. robustus*	
	Hap3-16S	100%	99.70%	NC_050328.1	*R. prolixus*	49<br>• 6 in SB2<br>• 7 in SB3<br>• 2 in SJ1<br>• 16 in SJ3<br>• 18 in T1
		78%	99.11%	AF045705.2	*R. robustus*	
	Hap4-16S	100%	99.53%	NC_050328.1	*R. prolixus*	2<br>• 2 in SB3
		78%	98.82%	AF045705.2	*R. robustus*	
	Hap5-16S	100%	98.83%	KC248984.1	*R. stali*	1<br>• 1 in T2
		78%	99.11%	AF045709.1	*R. pictipes*	
	Hap6-16S	100%	100%	KC248984.1,<br>KC248983.1	*R. stali*<br>*R. stali*	3<br>• 3 in T2
			99.70%	AY035437.1	*R. stali*	
	Hap7-16S	100%	98.60%	KC248984.1	*R. stali*	1<br>• 1 in SB3
		69%	99.33%	KT805174.1	*R. stali*	
	Hap8-16S	100%	98.83%	KC248984.1	*R. stali*	8<br>• 8 in SB3
		69%	99.67%	KT805174.1	*R. stali*	
28S-D2 **(578-bp)**	Hap1-D2	100%	100%	MW045637.1<br>AF435858.1	*R. robustus*<br>*R. robustus*	12<br>• 3 in C1<br>• 1 in C3<br>• 3 in R2<br>• 3 in G1<br>• 2 in G2
		100%	99.83%	AF435857.1<br>AF435860.1	*R. robustus*<br>*R. prolixus*	
		100%	99.65%	AF435859.1<br>JQ897670.1	*R. robustus*<br>*R. neglectus*	
	Hap2-D2	100%	99.65%	MW045637.1<br>AF435859.1<br>AF435858.1<br>JQ897670.1	*R. robustus*<br>*R. robustus*<br>*R. robustus*<br>*R. neglectus*	2<br>• 2 in C1
		100%	99.48%	AF435860.1<br>AF435857.1	*R. prolixus*<br>*R. robustus*	
	Hap3-D2	100%	99.65%	MW045637.1<br>AF435858.1	*R. robustus*<br>*R. robustus*	3<br>• 2 in C1<br>• 1 in C3
		100%	99.48%	AF435857.1<br>AF435860.1	*R. robustus*<br>*R. prolixus*	
		100%	99.31	AF435859.1	*R. robustus*	
	Hap4-D2	100%	99.48%	MW045637.1<br>AF435859.1<br>AF435858.1	*R. robustus*<br>*R. robustus*<br>*R. robustus*	5<br>• 2 in C1<br>• 1 in C2<br>• 1 in R1<br>• 1 in G2
		100%	99.31%	AF435857.1<br>AF435860.1	*R. robustus*<br>*R. prolixus*	
		100%	99.13%	MW045635.1<br>GQ853380.1	*Psammolestes coreodes*<br>*Psammolestes arthuri*	

All sequences with 100% cover and > 99% Identity with GenBank sequences are shown (cover < 100% also shown when Identity is low), giving their Access Number and the correspondent species. The number of individuals with each haplotypes and where they were captured are given. C: Cobija, G: Guayaramerín, R: Riberalta, SB: San Borja, SJ: San Joaquin, T: Trinidad.

Figure S2. Maximum likelihood tree constructed with the 17 *Cytb* haplotypes jointly with < 100% coverage sequences deposited in GenBank: 94 sequences belonging to 13 species (369-bp alignment). Tree constructed using IQ-Tree after determining the best substitution model using ModelFinder. UFBoot values based on 1000 repetitions are shown on the nodes by colors (green: > 95%, orange: 90-95%, red: 70-90%). SH-aLRT support (%)/ultrafast bootstrap support UFBoot (%) are shown for the principal nodes of interest. Species and subclades of *R. robustus* s.l. are differentiated by colors. *Triatoma infestans* sequence as out-group (Tinf). Species are: *R. barretti* (Rbar), *R. brethesi* (Rbre), *R. colombiensis* (Rcol), *R. ecuadoriensis* (Recu), *R. montenegrensis* (Rmon), *R. nasutus* (Rnas), *R. neglectus* (Rneg), *R. pallescens* (Rpal), *R. pictipes* (Rpic), *R. prolixus* (Rpro), *R. robustus* (Rrob) and its subclades (RrobI to RrobV), *R. stali* (Rsta), and *R. taquarussuensis* (Rtaq).

Notes: because of the shorter size of sequences, Hap_14 is identical to Hap_12, Hap_5 is identical to Hap_4, Hap_7 is identical to Hap_3, AF421341.1_RrobII is identical to Hap_11, KC249236.1_Rpic is identical to KC249235.1_Rbre, KT805172.1_Rsta is identical to Hap_16, AF045713.1_Rpic is identical to Hap_15, FJ887793.1_Rrob is identical to Hap_3, and KC249237.1_Rsta is identical to Hap_15.

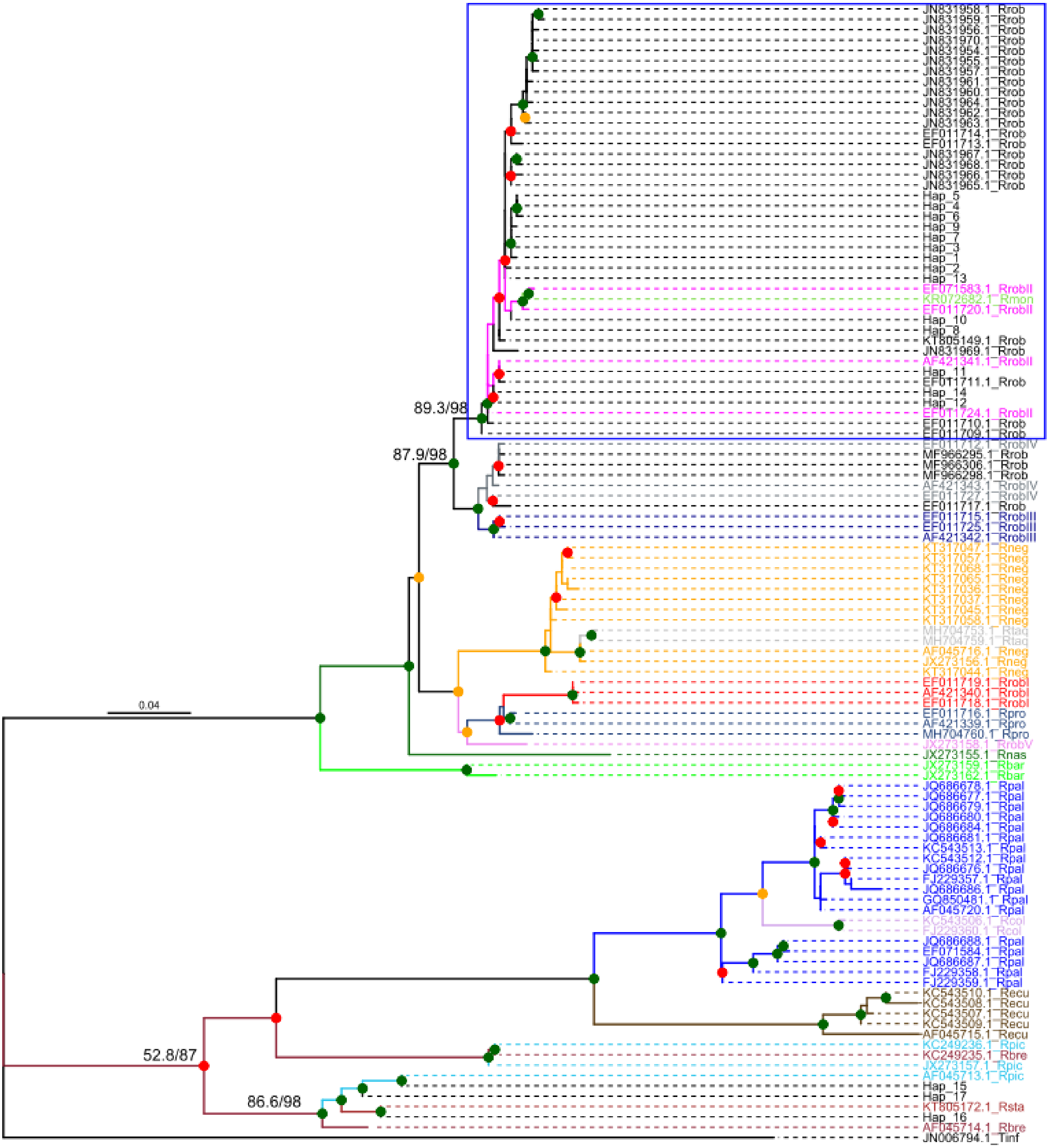

Figure S3. Maximum likelihood tree constructed with the eight 16S haplotypes jointly with sequences deposited in GenBank A) with 100%-coverage: 9 sequences belonging to 8 species (431-bp alignment), and B) with <100%-coverage: 20 sequences belonging to 9 species (274-bp alignment). Tree constructed using IQ-Tree after determining the best substitution model using ModelFinder. UFBoot values based on 1000 repetitions are shown on the nodes by colors (green: >95%, orange: 90-95%, red: 70-90%). SH-aLRT support (%)/ultrafast bootstrap support UFBoot (%) are shown for the principal nodes of interest. Species are differentiated by colors. *Triatoma infestans* sequence as out-group (Tinf). Species are: *R. brethesi* (Rbre), *R. colombiensis* (Rcol), *R. neglectus* (Rneg), *R. neivai* (Rnei), *R. pictipes* (Rpic), *R. prolixus* (Rpro), *R. robustus* (Rrob), and *R. stali* (Rsta).

Notes: in A: Hap_6 is identical to KC248983.1_Rsta. In B: Hap_6 is identical to AY035437.1_Rsta, EU827206.1_Rrob is identical to EU822954.1_Rpro, Hap_1 is identical to KT805173.1_Rrob, Hap_2 is identical to MF966358.1_Rrob, Hap_8 is identical to Hap_7, Hap_3 is identical to MF966358.1_Rrob, and Hap_4 is identical to MF966358.1_Rrob.

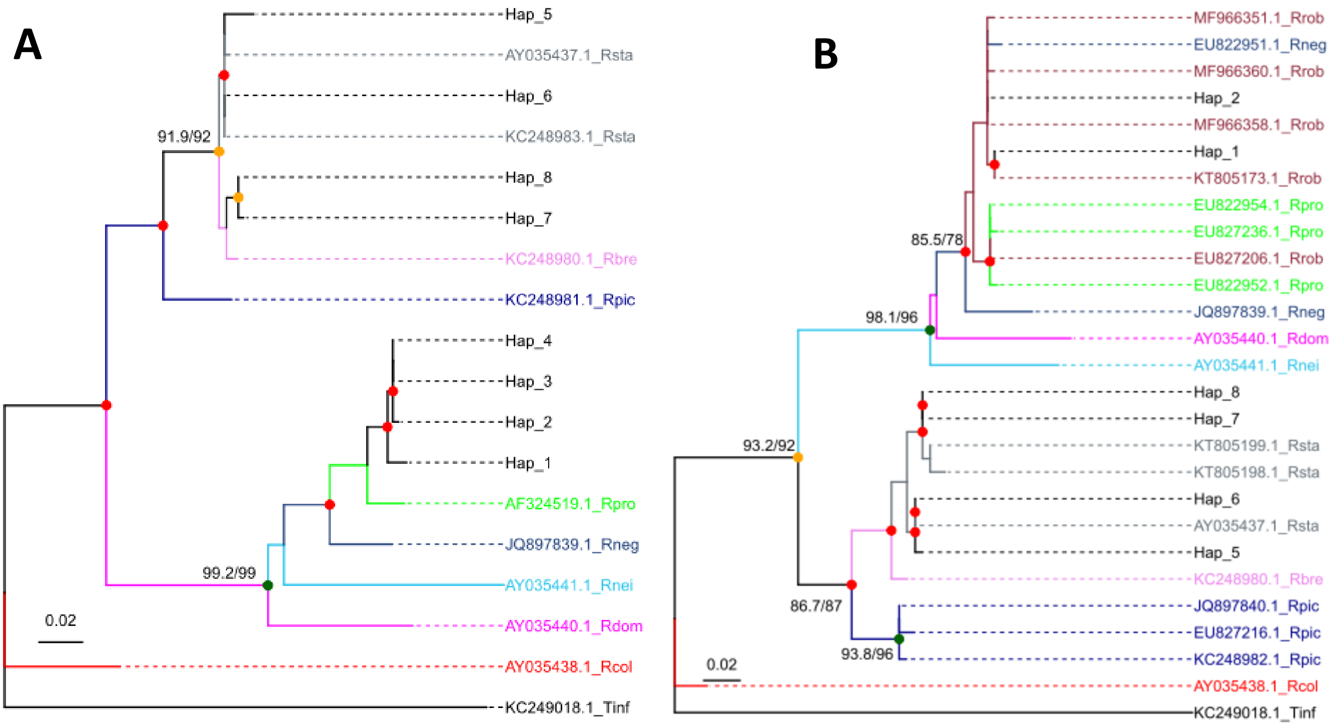

Figure S4. Maximum likelihood tree constructed with the four 28S D2 haplotypes jointly with sequences deposited in GenBank A) with 100%-coverage: 6 sequences belonging to 3 species (578-bp alignment), and B) with <100%-coverage: 21 sequences belonging to 9 species (399-bp alignment). Tree constructed using IQ-Tree after determining the best substitution model using ModelFinder. UFBoot values based on 1000 repetitions are shown on the nodes by colors (green: >95%, orange: 90-95%, red: 70-90%). SH-aLRT support (%)/ultrafast bootstrap support UFBoot (%) are shown for the principal nodes of interest. Species are differentiated by colors. *Triatoma infestans* sequence as out-group (Tinf). Species are: *R. colombiensis* (Rcol), *R. ecuadoriensis* (Recu), *R. nasutus* (Rnas), *R. neglectus* (Rneg), *R.* pallescens (Rpal), R. *pictipes* (Rpic), *R. prolixus* (Rpro), *R. robustus* (Rrob), and *R. stali* (Rsta).

Notes: in A: AF435861.1_Rrob is identical to AF435862.1_Rpro, and Hap_1 is identical to AF435858.1_Rrob. In B: KY111699.1_Rsta is identical to JQ897671.1_Rpic, AF435861.1_Rrob is identical to AF435862.1_Rpro, MF966325.1_Rrob is identical to MF966329.1_Rrob, Hap_2 is identical to KY111671.1_Rrob, KC543526.1_Rpal is identical to KC543518_Recu, AF435858.1_Rrob is identical to AF435862.1_Rpro, and Hap_1 is identical to AF435862.1_Rpro.

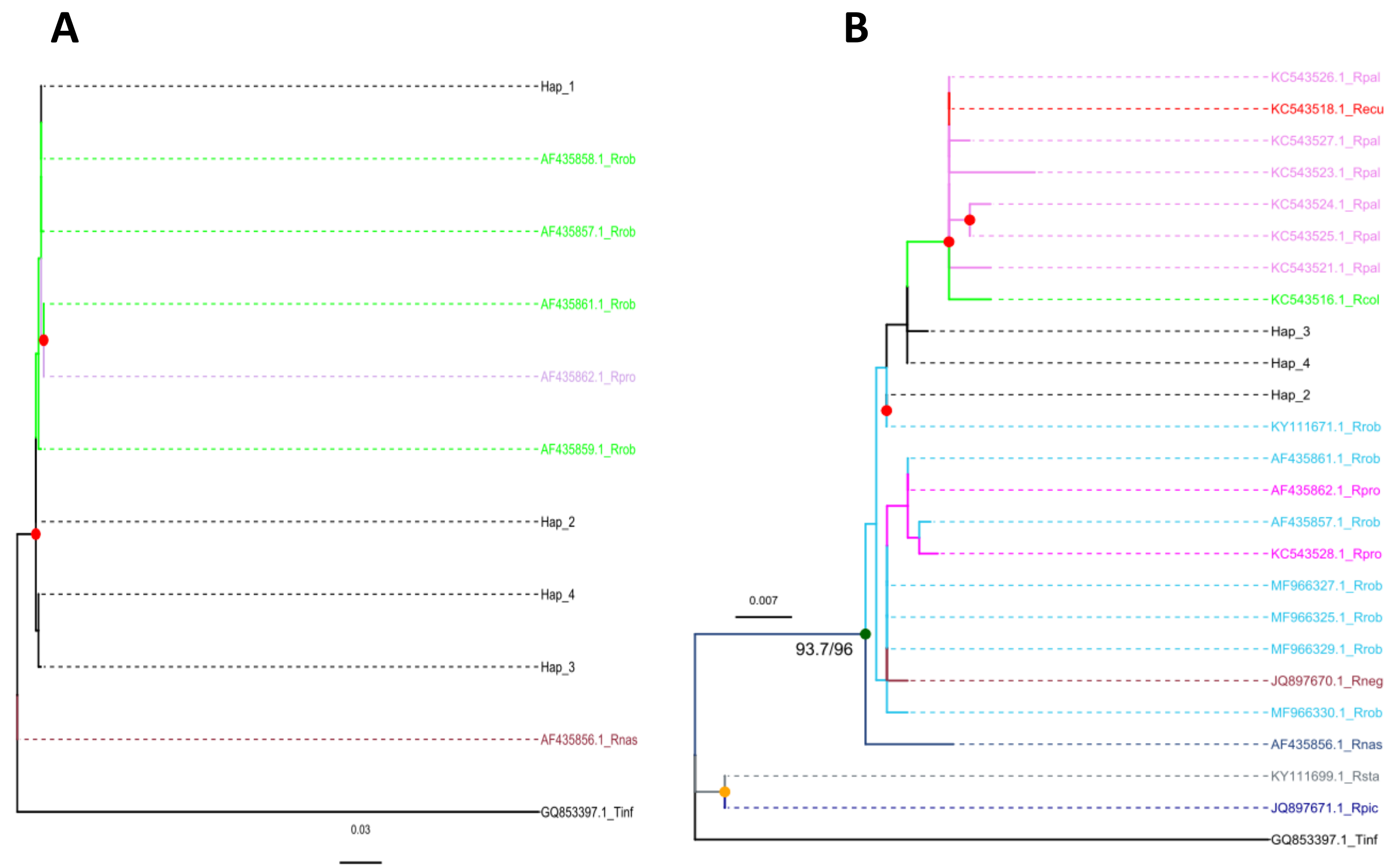

Figure S5. Agarose gel electrophoresis (3% agarose) of PCR amplified products using mini-exon multiplex PCR (MMPCR). Lanes 3, 4, 6, 9, 11, 12, and 15 are examined triatomes from study locations, San Borja 3 (SB3), Trinidad 1 (T1), and San Joaquin 3 (SJ3). Lanes 1, 2, 8, 13, and 16 are PCR results from DNAs of different well known strains of *T. cruzi*, and lanes 5 and 17 from *T. rangeli* reference strain. Lanes 10 and 18 are PCR control with water as template. Interpretation of corresponding *Trypanosoma* species and *T. cruzi* DTUs were based on previous data: 200-bp for TcI, 250-bp for TcII-TcV-TcVI, 150-bp for TcIII-TcIV, and 100-bp for *T. rangeli* (Aliaga et al., 2011).

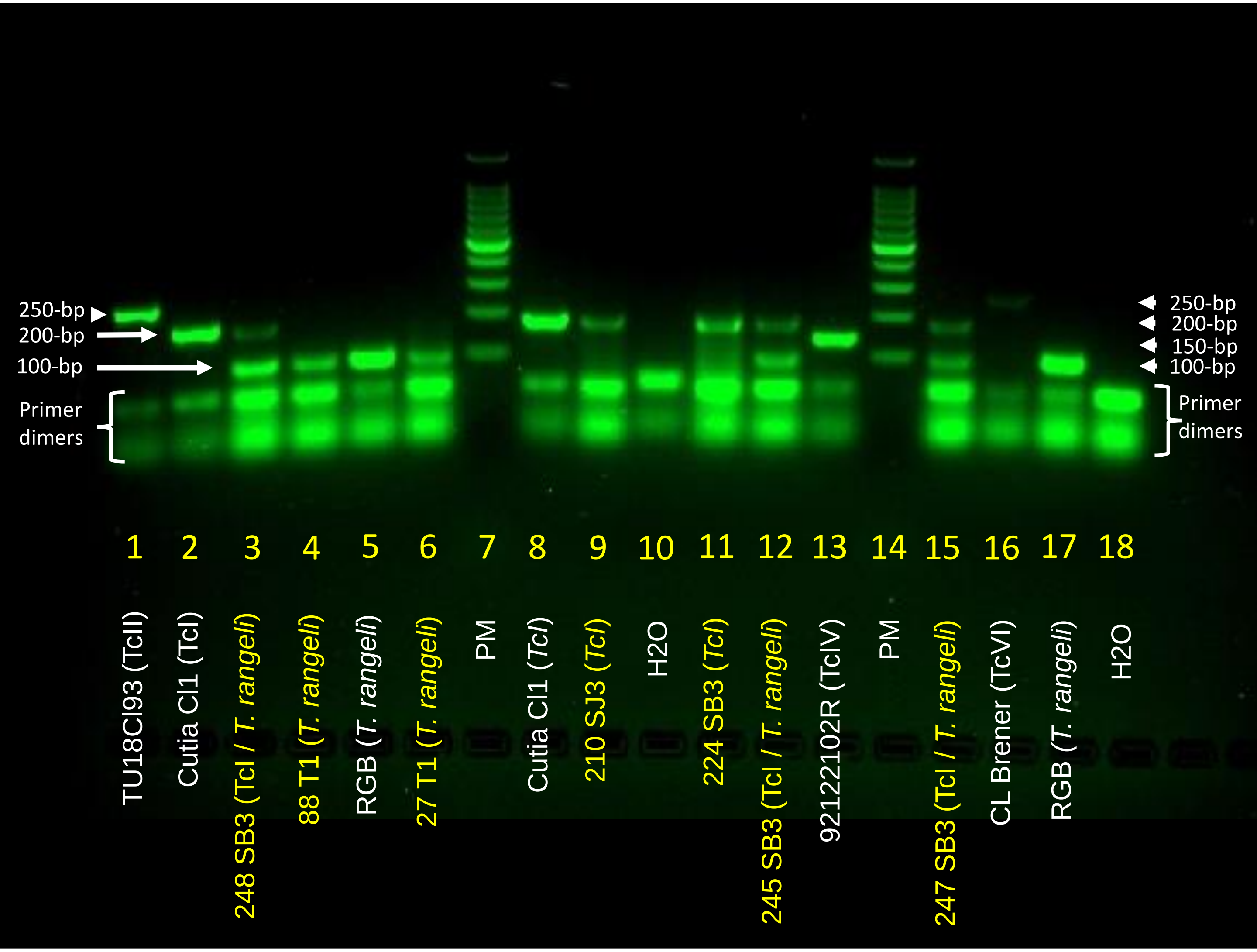

Supplement: Supplementary file 1 — Additional file 1: Text S1. Methodology used for the different molecular characterization of Triatominae, blood source meal and Trypanosoma. Table S1. Description of primers used in molecular characterization of triatomines, blood source meals and Trypanosoma. Table S3. Distribution of the insects by capture points and developmental stages. Figure S1. a Environmental description of capture points in Trinidad, San Joaquin and San Borja. b Environmental description of capture points in Riberalta, Guayaramerín and Cobija. Table S4. General description of the environment around each capture point inside a circle of 500 m in radius. Table S5. Description of the haplotypes found for the Cytb, 16S, and 28S-D2 gene fragments. Figure S2. Maximum likelihood tree constructed with the 17 Cytb haplotypes jointly with < 100% coverage sequences deposited in GenBank. Figure S3. Maximum likelihood tree constructed with the eight 16S haplotypes jointly with sequences deposited in GenBank. Figure S4. Maximum likelihood tree constructed with the four 28S-D2 haplotypes jointly with sequences deposited in GenBank. Figure S5. Agarose gel electrophoresis (3% agarose) of PCR-amplified products using mini-exon multiplex PCR (MMPCR). [file 13071_2022_5423_MOESM1_ESM.pdf]
